# Chronotopic maps in human supplementary motor area

**DOI:** 10.1371/journal.pbio.3000026

**Published:** 2019-03-21

**Authors:** Foteini Protopapa, Masamichi J. Hayashi, Shrikanth Kulashekhar, Wietske van der Zwaag, Giovanni Battistella, Micah M. Murray, Ryota Kanai, Domenica Bueti

**Affiliations:** 1 International School for Advanced Studies (SISSA), Trieste, Italy; 2 Global Center for Medical Engineering and Informatics, Osaka University, Suita, Japan; 3 School of Psychology, University of Sussex, Brighton, United Kingdom; 4 Animal Imaging and Technology, Ecole Polytechnique Fédérale de Lausanne, Center for Biomedical Imaging (CIBM), Lausanne, Switzerland; 5 Spinozisme Centre for Neuroimaging, Royal Academy for Arts and Sciences, Amsterdam, the Netherlands; 6 Department of Radiology, Centre Hospitalier Universitaire Vaudois (CHUV), University of Lausanne, Lausanne, Switzerland; 7 Department of Neurology, Memory and Aging Center, University of California San Francisco, San Francisco, California, United States of America; 8 The Laboratory for Investigative Neurophysiology (The LINE), Department of Radiology and Department of Clinical Neurosciences, University Hospital Center and University of Lausanne, Lausanne, Switzerland; 9 The EEG Brain Mapping Core, Centre for Biomedical Imaging (CIBM), Lausanne, Switzerland; 10 The Ophthalmology Service, Fondation Asile des Aveugles and University of Lausanne, Lausanne, Switzerland; 11 Department of Hearing and Speech Sciences, Vanderbilt University, Nashville, Tennessee, United States of America; 12 Sackler Centre for Consciousness Science, University of Sussex, Brighton, United Kingdom; 13 Araya, Inc., Tokyo, Japan; McGill University, CANADA

## Abstract

Time is a fundamental dimension of everyday experiences. We can unmistakably sense its passage and adjust our behavior accordingly. Despite its ubiquity, the neuronal mechanisms underlying the capacity to perceive time remains unclear. Here, in two experiments using ultrahigh-field 7-Tesla (7T) functional magnetic resonance imaging (fMRI), we show that in the medial premotor cortex (supplementary motor area [SMA]) of the human brain, neural units tuned to different durations are orderly mapped in contiguous portions of the cortical surface so as to form chronomaps. The response of each portion in a chronomap is enhanced by neighboring durations and suppressed by nonpreferred durations represented in distant portions of the map. These findings suggest duration-sensitive tuning as a possible neural mechanism underlying the recognition of time and demonstrate, for the first time, that the representation of an abstract feature such as time can be instantiated by a topographical arrangement of duration-sensitive neural populations.

## Author summary

Sensing the passage of time is a common experience of our everyday life activity. Even without a watch, we can, for example, tell whether the bus we are waiting for is late. The neuronal mechanism that enables us to sense the passage of time is largely unknown. Here, we asked healthy human volunteers to discriminate between visual events of varying durations while we measured brain activity via functional magnetic resonance imaging (fMRI). The results show that distinct portions of the supplementary motor area (SMA)—a region of the cerebral cortex important for both motor preparation and time perception—respond preferentially to different durations. The portions of the SMA responding to similar durations are in close spatial proximity on the cortex, and their response is greater for preferred and neighboring durations and suppressed for distant ones. The spatial arrangement of duration-selective portions of the SMA could be the mechanism that enables us to efficiently sense that a certain duration has elapsed.

## Introduction

Time is a particularly elusive dimension of everyday experiences. We cannot see or touch time; nevertheless, we clearly sense its flow and adjust our behavior accordingly. When dancing, our body entrains to the musical tempo. Even without a watch, we can detect when the bus we are waiting for is late.

While a growing body of evidence highlights the contribution of many different brain regions to temporal computations, the neuronal mechanisms underlying our capacity to perceive time remain largely unknown [1][2].

Single-neuron recordings in animals suggest that the encoding of temporal information in the millisecond/second range is achieved via duration-tuned mechanisms [3][4][5]. Duration-selective cells have been observed in cats’ early visual cortex [5], in cats’ and bats’ primary auditory cortex [6][7], and, more recently, in monkeys’ medial premotor and prefrontal cortices [3][4][8]. In the human brain, the existence of such mechanisms has been recently suggested by psychophysical studies [9][10] and by a single neuroimaging experiment [11]. Psychophysical studies show that the repeated presentation of a visual stimulus or an auditory rhythm of a given duration (i.e., “adaptor”) affects the perceived duration of a subsequent visual stimulus or rhythm (i.e., “after-effect”). After-effects are stronger if the temporal distance between the “adaptor” and the judged stimulus is optimal, suggesting the existence of tuning profiles [9][10] for which the selectivity is highest for the preferred duration (PD) and slowly decays with distance from it. Duration adaptation has also been shown to influence the activity of the inferior parietal lobule (IPL) in the human brain. Neural activity in the IPL is suppressed for stimuli of the same duration and enhanced for stimuli of different durations [11].

However, previous studies in either the animal or the human brain have not clarified whether neurons tuned to different durations have an ordered topographical arrangement in duration-sensitive areas of the brain. Whether this ordered arrangement is a specific property of single or multiple brain regions also remains unknown.

Neuronal tuning and topography are mechanisms widely used in the brain to represent sensory information [12][13], including abstract features like quantities [14]. Showing the existence of a temporal topography could therefore be very important to gain insights on the computational architecture underlying time perception and to link the representation of time to that of other sensory features, such as stimulus orientation.

## Results

**Fig 1:**
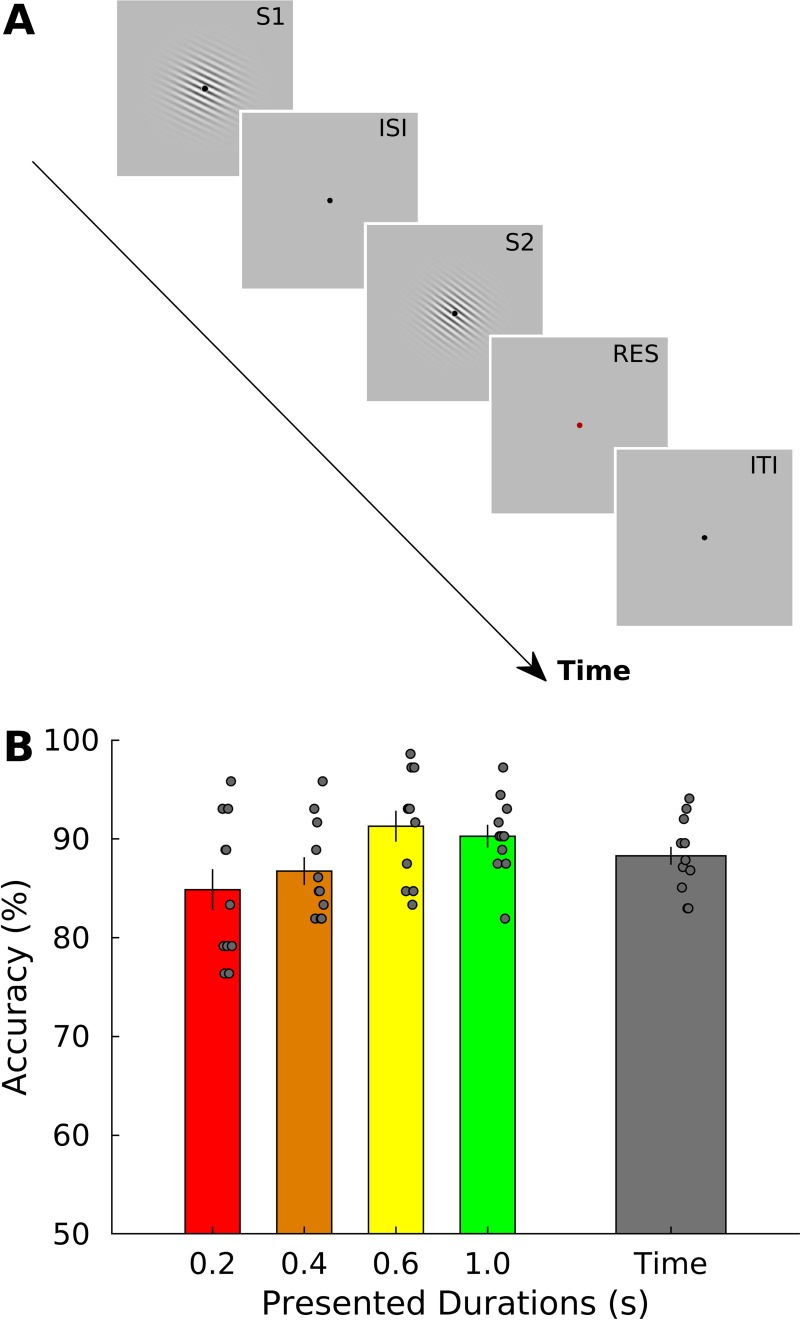
Stimulus sequence and behavioral results of Exp 1. (A). Schematic representation of the stimulus sequence in a trial of Exp 1. In each trial, a standard (S1) and a comparison duration (S2) were presented in sequence. S1 could be one of four different durations (0.2, 0.4, 0.6, and 1 s). S2 could be either shorter or longer than S1 (Weber ratio was set to 0.4). Stimuli were sinusoidal Gabor patches varying in orientation. Orientation changes were task irrelevant. Participants were asked, by pressing one of two response keys, to judge whether the duration of S2 was shorter or longer than S1. (B) Group average (*N* = 11) of percentage of accuracy in the time task plotted separately for each of the four durations and as a mean of them (“overall accuracy,” rightmost bar; the gray dots are the different subjects). The data can be found in S1 Data. Error bars are standard errors. S1, first stimulus; S2, second stimulus.

To examine if chronotopic representations exist in the human brain, we used ultrahigh-field functional magnetic resonance imaging (fMRI) at 7-Tesla (7T) in two distinct experiments. In the first of these experiments (Exp 1), we measured brain activity while participants (*N* = 11) decided whether the second stimulus (S2) of a pair was longer or shorter than the first stimulus (S1; see Fig 1A). In this experiment, we used four different duration ranges (i.e., S1 = 0.2, 0.4, 0.6, and 1 s). Stimuli were visual gratings (i.e., Gabor patches) varying in both orientation and duration. Orientation changes were task irrelevant (see Materials and methods for details).

Behavioral data indicate that participants performed equally well in all tested durations (see Fig 1B and S1 Data). Proportion of correct responses for each S1 duration condition (i.e., 0.2, 0.4, 0.6, and 1 s) were 85.1% ± 7.1% (mean ± standard deviation), 87.0% ± 4.9%, 91.5% ± 5.4%, and 90.6% ± 4.1%, respectively. Overall accuracy was 88.6% ± 3.7%. Although a one-way repeated measures ANOVA with within-subject factor of S1 durations showed a significant main effect (F_3,30_ = 4.824, *P* < 0.05), pairwise posthoc tests showed no significant difference between the different combinations of S1 durations (all *P* values > 0.05, Bonferroni corrected for multiple comparisons).

For the analysis of Exp 1, we used a mass-univariate General Linear Model (GLM) approach. We used separate regressors for each of the four different duration ranges. These regressors of interest modeled the offsets of the S1 duration and were convolved with the canonical hemodynamic response function (HRF; see Materials and methods for more details about the modeling of regressors of no interest). We used event offset because it was the moment when the duration of a stimulus became available to participants.

We first identified the regions associated with the presentation of the four S1 durations together. As expected from previous neuroimaging findings [15][16], these regions were visual, parietal, and frontal cortices (see S1 Fig and S1 Table).

We then focused on the identification of the brain regions that were maximally activated for each specific S1 duration and that clearly showed a topographical arrangement of duration-selective voxels.

**Fig 2:**
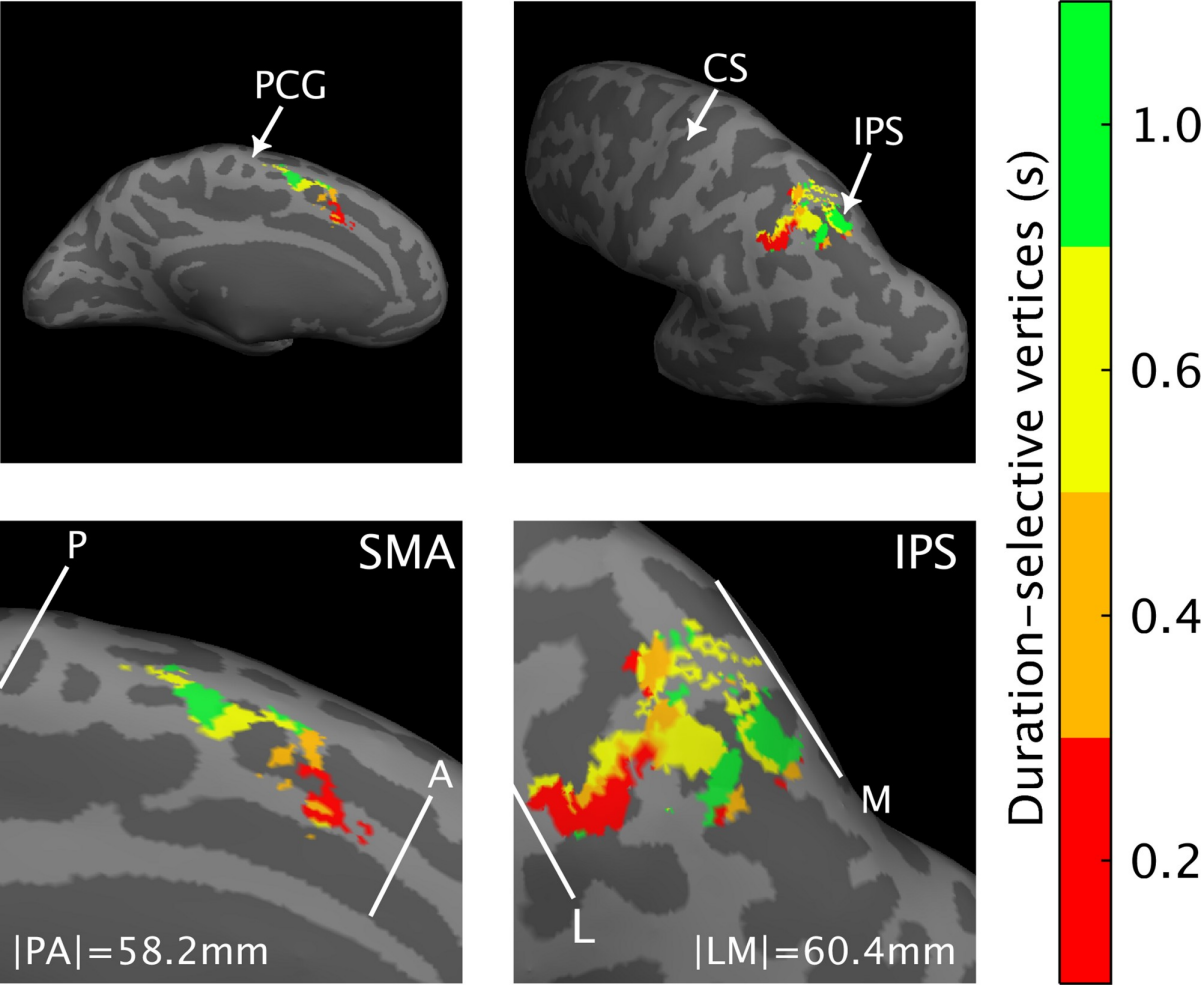
Group-level fMRI results of Exp 1. M and L view of the left hemisphere, with the group-level statistical results (*N* = 11) overlaid on the inflated Dartel-11 template. The figure shows the cluster of vertices (i.e., voxels projected onto the brain surface) classified according to a winner-take-all procedure based on statistical t-maps, as maximally responsive to each of the four S1 durations (0.2, 0.4, 0.6, and 1 s). Each color codes a different label; the color scale goes from red (shortest S1) to green (longest S1). Statistical threshold for t-maps was set to *P*_FWE_ < 0.05 cluster-level corrected for multiple comparisons across the whole brain. Duration-selective vertices were found in SMA (leftward panel) but also in the IPS. The durations of the color bar are red = 0.2, orange = 0.4, yellow = 0.6, and green = 1 s. The white lines give an example of the map borders, as they were drawn to estimate the wRD from either the P or the L border in individual subjects. For the wRD computation, though, borders were drawn in each individual subject in their native space. The size of the maps reported in the figure is the average size calculated across the 11 subjects when their map was in a common Dartel space. The data can be found in S2 Data. A, anterior; CS, central sulcus; fMRI, functional magnetic resonance imaging; FWE, familywise error; IPS, intraparietal sulcus; L, lateral; M, medial; P, posterior; PCG, precentral gyrus; S1, first stimulus; SMA, supplementary motor area; wRD, weighted relative distance.

Fig 2, upper panel shows the group-level significant clusters computed for each of the four duration ranges in the temporal task (*P*_FWE_ < 0.05, cluster level corrected for multiple comparisons across the whole brain; see also S2 Data). Each color codes the cluster of voxels that was classified according to a winner-take-all procedure based on t-statistic maps, as maximally responsive to each of the different duration ranges. The color scale ranges from red, corresponding to voxels responsive to the shortest duration (0.2 s), to green, the voxels maximally responsive to the longest duration (1 s).

As indicated by the gradual change of color in Fig 2, we found a topographic organization of duration-sensitive voxels in the supplementary motor area (SMA; see leftward panels) and in part of the intraparietal sulcus (IPS, see rightward panels) of the left hemisphere. In SMA, this progression was in the rostro–caudal direction, with voxels sensitive to the shortest duration located in the anterior SMA and those sensitive to the longest duration in the posterior part.

In the IPS, the progression was in the lateral–medial direction i.e., voxels maximally responsive to the shorter duration were closer to the lateral border of the map compared to those sensitive to the longer duration.

To quantitatively assess the spatial distribution of duration-selective voxels in SMA and IPS during the temporal discrimination task, we analyzed both volumetric and surface data of each individual subject (see Materials and methods for details).

At the surface level, for each subject and each duration-selective cluster of vertices (i.e., voxels projected onto the brain surface), we calculated the weighted relative distance (wRD) from the posterior and the lateral borders of the chronomap for SMA and IPS, respectively (see Materials and methods for details). Borders of the maps were identified in each individual subject in their native space.

**Fig 3:**
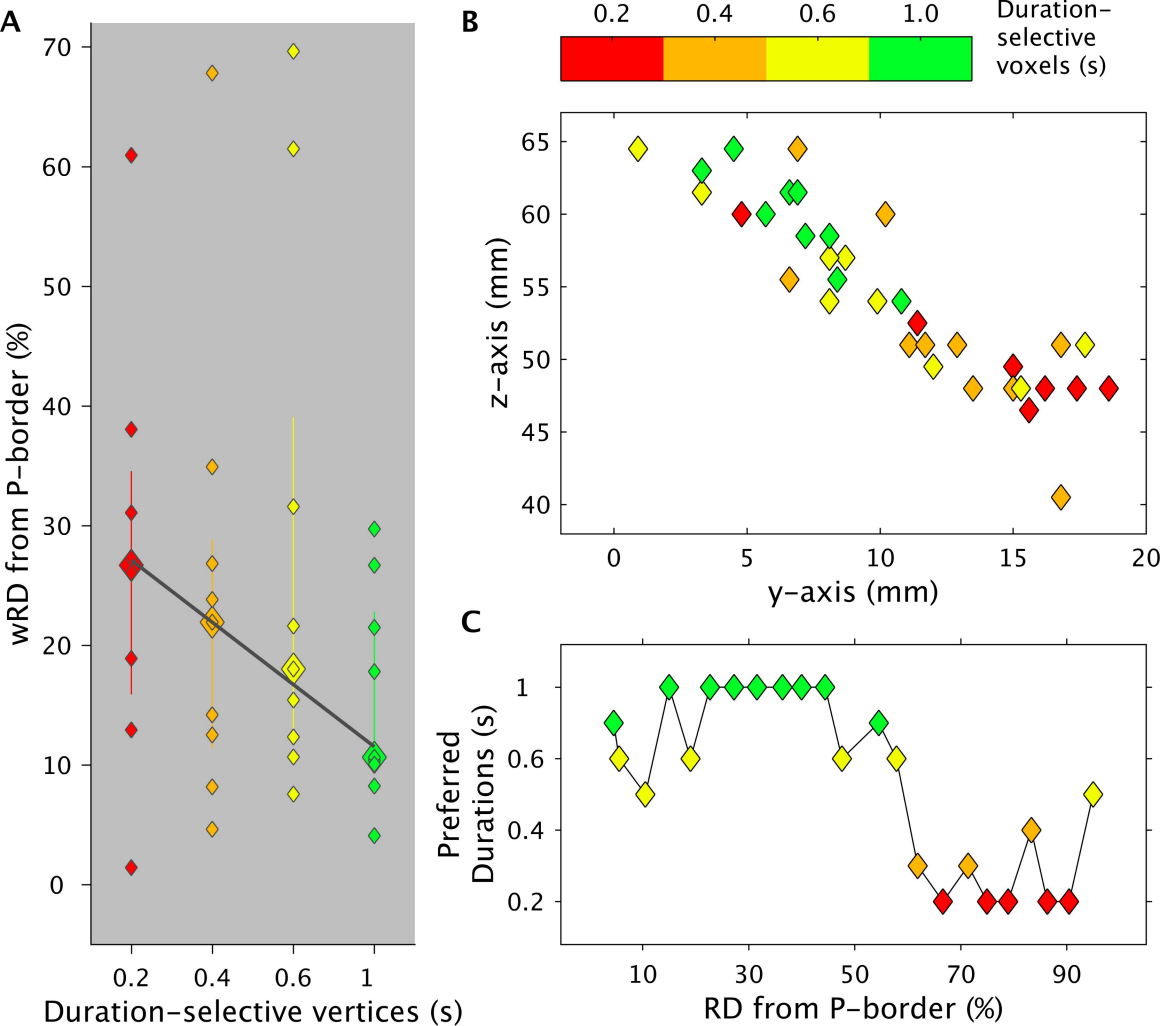
Spatial progression of SMA chronomaps in Exp 1. Panel (A) shows for each duration-selective vertex the group median (biggest colored diamonds), the full distribution of individual data (smaller diamonds), and the fitted slope of the wRD from the P border of the chronomap. wRD were first computed for each individual subject on chronomaps overlaid on flattened surfaces in participant’s native space. The P border was chosen to be the most posterior part of the precentral gyrus. (B) wCntrs for duration-selective voxels in SMA. 2D projection of wCntrs in the y-z plane. wCntrs are color coded according to duration selectivity. The color scale goes from red (shortest S1 = 0.2 s) to green (longest S1 = 1 s). Different colors indicate voxels with different duration selectivity; diamonds with the same color represent the different subjects (*n* = 11). This value differs across duration conditions because not all subjects had the full range of duration-selective voxels. (C) Group average of preferred durations (y-axis) of voxels lying at different distances (in x-axis, RD) from the P border of the chronomaps. The data can be found in S1 Data. P, posterior; RD, relative distance; SMA, supplementary motor area; wCntr, weighted centroid; wRD, weighted relative distance.

Fig 3A (see also S1 Data) shows in the left SMA for each duration-selective cluster the full distribution of individual wRD and their median value. In the figure, it is also displayed a fitted slope based on these median values (for individual maps and individual slopes, see S2 Fig and S3 Fig and S4 and S3 Data).

The plot shows, as expected from the visual inspection of the group-level brain map, that the distance from the posterior border of the SMA is longer for vertices responsive to the shortest duration (0.2 s) and becomes progressively shorter for vertices responsive to the longer duration range. This progression was also present in the majority of the subjects (for individual maps, see S2 Fig and S4 Data), as revealed by the statistically significant analysis of the wRD slopes (Wilcoxon rank sum test on individual slopes, for which we tested the existence of a negative slope versus a slope equal to zero, *P* = 0.017; for a better appreciation of individual slopes, see also S3 Fig and S3 Data).

To confirm the spatial progression of SMA chronomap, we also identified for each individual volumetric map the duration preferred by the majority of the activated voxels that laid at different distances from the posterior border of the chronomap (individual chronomaps in the subjects’ native space were parceled in volumetric bins of 1.5-mm width; for details, see Materials and methods). The relative distance from the posterior border of these preferred durations is shown in Fig 3C and S1 Data. As seen previously, the shorter the distance from the posterior border, the greater the number of voxels preferring the longer duration ranges (diamonds in colder colors). The greater the distance from the posterior border, the greater the number of voxels preferring the shorter duration ranges (diamonds in warmer colors). A similar result is shown in Fig 3B and S1 Data, in which we plot for each subject the weighted centroids (wCntrs) of each duration-selective cluster (centroids were computed on data normalized to a common Dartel space). Within the SMA, the centroids of the shortest-duration–selective cluster (red diamonds) were generally located anteriorly compared to the centroids of the longest-duration–selective cluster (green diamonds).

In the IPS, the topographical arrangement of voxels (i.e., from lateral to medial for short to long durations) was apparent at the group level, but it was less consistently observed at the single-subject level (for individual maps, see S4 Fig and S4 Data). Indeed, only five out of 11 subjects showed the appropriate spatial distribution of duration-selective voxels. Moreover, when we looked at the wRD, there was no statistically significant effect of the slope (Wilcoxon test *P* = 0.737; see S3 Fig for individual slopes; see S5 Fig and S3 Data for preferred durations and centroids).

**Fig 4:**
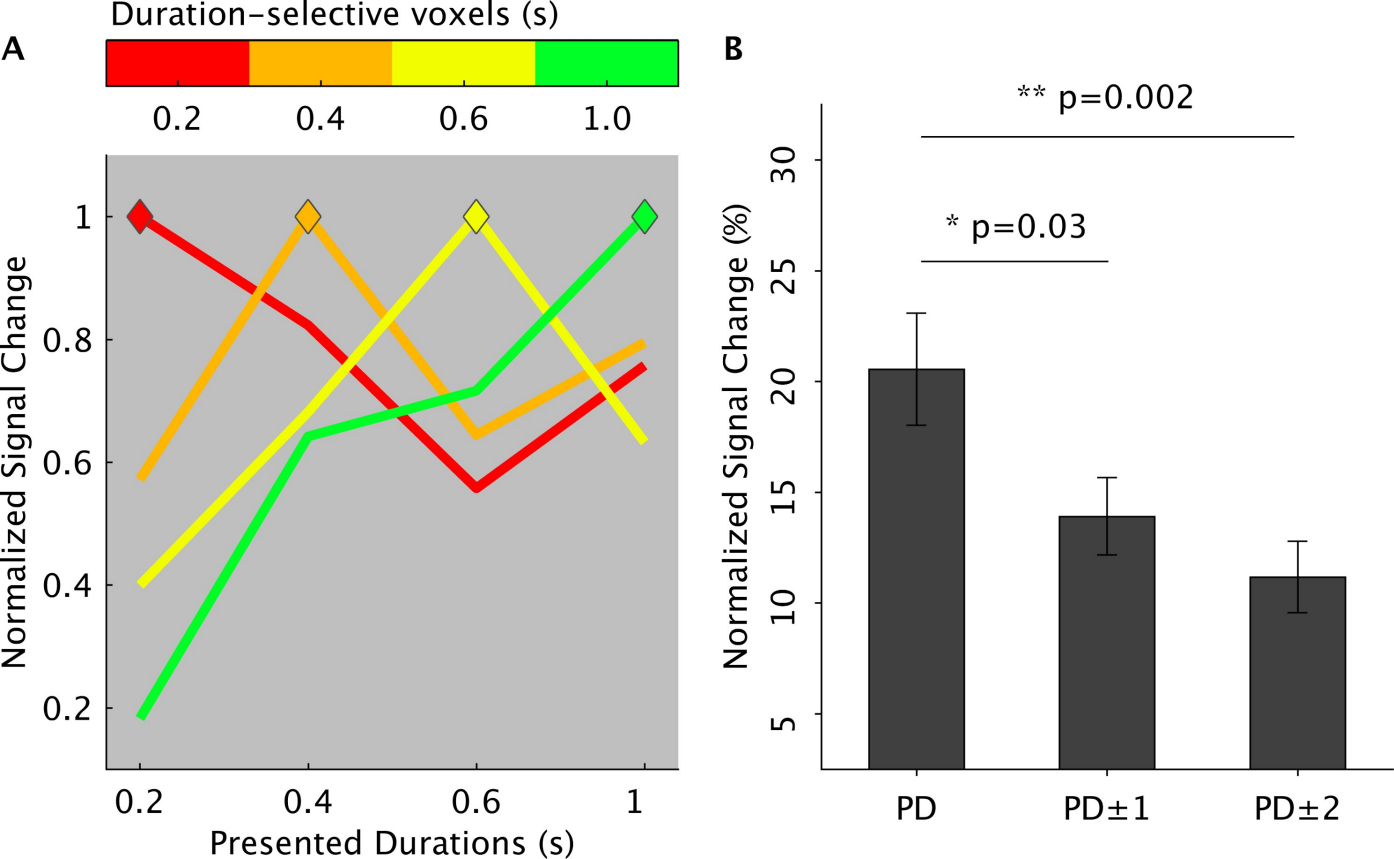
Duration tuning of Exp 1. (A) Group average of normalized BOLD responses (y-axis) of duration-selective voxels (different lines are different duration-selective voxels) for preferred and nonpreferred durations. On the x-axis are the four presented durations. The BOLD signal in the duration-selective voxels is aligned to the presentation timings of the different duration ranges (i.e., second volume after S1 offset). The colored diamonds represent the point in time when the hemodynamic response of duration-selective voxels matched the presentation timing of the appropriate duration (e.g., red-labeled voxels when the shortest S1 duration is presented). The color code is as in Fig 2. Normalization was performed first in each individual subject to the mean signal intensity across fMRI runs and then for each duration-selective cluster to the signal associated to the PD. (B) Normalized BOLD response to PDs, neighboring (PD ±1), and distant durations (PD ± 2) averaged across subjects and duration-selective voxels. The data can be found in S1 Data. Error bars are standard errors. BOLD, blood oxygenation level-dependent; fMRI, functional magnetic resonance imaging; PD, preferred duration; S1, first stimulus.

To examine the response tuning of the voxels to a given duration range, we looked at the change of the hemodynamic response of these voxels for nonpreferred durations. Fig 4 (S1 Data) shows the hemodynamic response of duration-sensitive voxels for the left SMA. In each duration-selective cluster, the hemodynamic response was normalized to the preferred duration (PD), i.e., the duration to which the cluster was maximally responsive to, based on t-statistics maps. As shown in panel A, for all duration-selective clusters (i.e., colored lines), we observed a modulation of the presented durations on the blood oxygenation level-dependent (BOLD) response. Specifically, the hemodynamic response, which peaked during the presentation of the PD (see the diamonds in the plot), slowly decayed for durations distant from the preferred one (PD versus PD ± 1, *P* < 0.03; PD versus PD ± 2, *P* < 0.002).

A similar result was obtained by plotting the normalized signal change of the shortest and longest duration-selective clusters over the trial period, when either a shortest or a longest duration was presented (see S6 Fig and S3 Data). As expected, after the stimulus offset, the hemodynamic response rose at a similar time in the two clusters, for the two presented durations (second TR after stimulus offset). However, the signal had a greater amplitude for the appropriate pair of stimulus and duration-selective cluster, e.g., the 0.2-s duration-selective cluster when a 0.2-s stimulus was presented.

Similar results were obtained in the IPS (S7 Fig and S3 Data) where the BOLD response was enhanced for preferred (PD) and neighboring (PD ±1) durations (PD versus PD ±1, *P* < 0.009) and suppressed for durations far (PD ± 2) from the preferred one (PD versus PD ± 2, *P* < 0.005).

**Fig 5:**
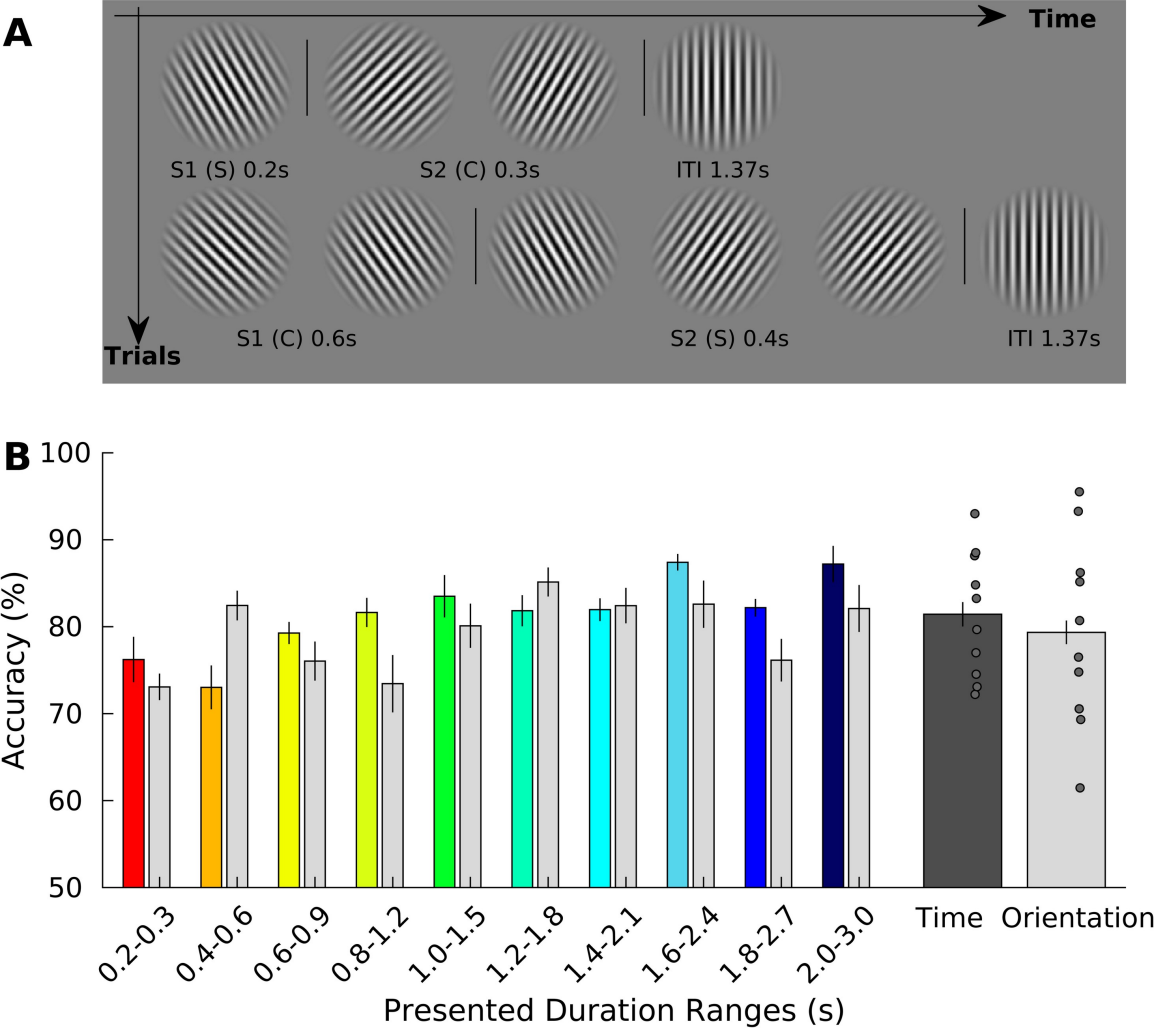
Stimulus sequence and behavioral results of Exp 2. (A) Schematic representation of the stimulus sequence in a trial (Exp 2). Within a trial, the sequence of orientation changes was fixed and was always leftward first, rightward second, and vertical last. Within the two “main orientations” (left and right), the grating continuously changed its orientation at a rate of 5 Hz, and the range of changes was between 30° and 45°. Participants were asked to discriminate which of the two “main orientations” (leftward or rightward) was displayed for longer time (time task) or to judge which one of them underwent the biggest change (orientation task). S is standard and C the comparison duration. There were 10 standard durations, ranging from 0.2 to 2 s, in step of 0.2 s. The presentation order of S and C was randomized and counterbalanced across trials. In half of the trials, S1 was a standard; in the other half, it was a comparison duration. The comparison duration was 50% of the standard. The vertical orientation signaled the time to make the response (by pressing one of two response keys on a keypad), and it was also the intertrial interval (1.37 s). (B) Average percentage of accuracy (*N* = 10) in the time and orientation task plotted separately for each of the 10 pairs of durations and as a mean of them (rightmost plot for time and orientation task). The data can be found in S1 Data. Error bars represent standard errors. Gray dots are different subjects. C, comparison duration; S, standard duration; S1, first stimulus.

In order to assess the robustness of Exp 1’s results, we ran an additional experiment (Exp 2, *N* = 10) in which we used a similar temporal discrimination task of visual stimuli (i.e., participants judged which of the two successive visual stimuli [S1 and S2] lasted longer). Visual stimuli were Gabor patches changing in orientation (see Fig 5A). In Exp 2, we introduced three main changes compared to Exp 1.

First, we used a broader range of durations, spanning from 0.2 to 3 s. Second, we used a method of stimulus presentation that was highly regular, i.e., different duration ranges were presented sequentially. We used pairs of stimuli (S1 and S2) varying in duration. In different pairs, we tested different duration ranges, e.g., S1 = 0.2 versus S2 = 0.3 s in one pair and S1 = 0.4 versus S2 = 0.6 s in a different pair (see Fig 5A). In each pair, we had a standard (T) and a comparison duration (T + ΔT); in half of the trials, the standard duration was S1, and in the other half, it was S2. The pairs were presented in a sequential manner, as to form cycles (i.e., a cycle is a series of trials [*N* = 10] for which we tested 10 duration ranges). In ascending cycles, we progressed from the shortest to the longest pair of stimuli; in descending cycles, it was the opposite. This design allowed us to evaluate whether there was a gradual spatial shift in cortical activation as the stimulus duration changed. Third, in addition to the temporal discrimination task, participants performed a nontemporal task for which they judged the angular orientation of the same visual gratings. This task was included to evaluate the task dependency of chronotopic representations.

In Exp 2, S1 and S2 stimuli were defined by different orientations (see Fig 5A and Materials and methods for full details of the tasks). S1 was leftward and S2 was rightward oriented. While keeping their main orientation, both S1 and S2 slightly changed their angular orientation. In the temporal task, participants judged which stimulus orientation was maintained for longer time, whereas in the orientation task, they judged which orientation underwent the biggest angular change.

Behavioral data inside the MRI scanner did not reveal any significant performance differences across the different durations (see Fig 5B and S1 Data, main effect of duration F_9_ = 1.303, *P* = 0.289) and the two tasks (main effect of task F_1_ = 0.309, *P* = 0.592; interaction effect F_1,9_ = 0.539, *P* = 0.842).

At the brain level, based on Exp 1 results, we focused on the identification of chronomaps in both SMA and IPS (for the details on the two regions of interest [ROI], see Materials and methods).

The cyclical presentation of the events, the very short stimulus onset asynchrony (SOA), together with the absence of jittering in interstimulus and intercycle intervals made this design particularly suitable for the population Receptive Field (pRF) method of analysis. pRF is an fMRI method of data analysis that is used to map response selectivity to any type of stimulus feature (e.g., the spatial position of a visual object [17][18]). The idea behind pRF is that neuronal receptive fields are a form of tuning functions. As pRF model, we used a one-dimensional Gaussian curve with two parameters: μ, the stimulus duration, and σ, the spread of the Gaussian. For the pRF modeling, we used the offset of all S1 durations, no matter whether S1 was a standard or a comparison duration. This procedure led to the identification of 17 durations (ranging from 0.2 to 3 s). For each time point of the fMRI timeseries, the overlap between the Gaussian tuning models and the presented stimulus profiles were estimated (see Material and methods for more details). The combination of Gaussian tuning models and the presented stimulus profiles were convolved to HRF.

**Fig 6:**
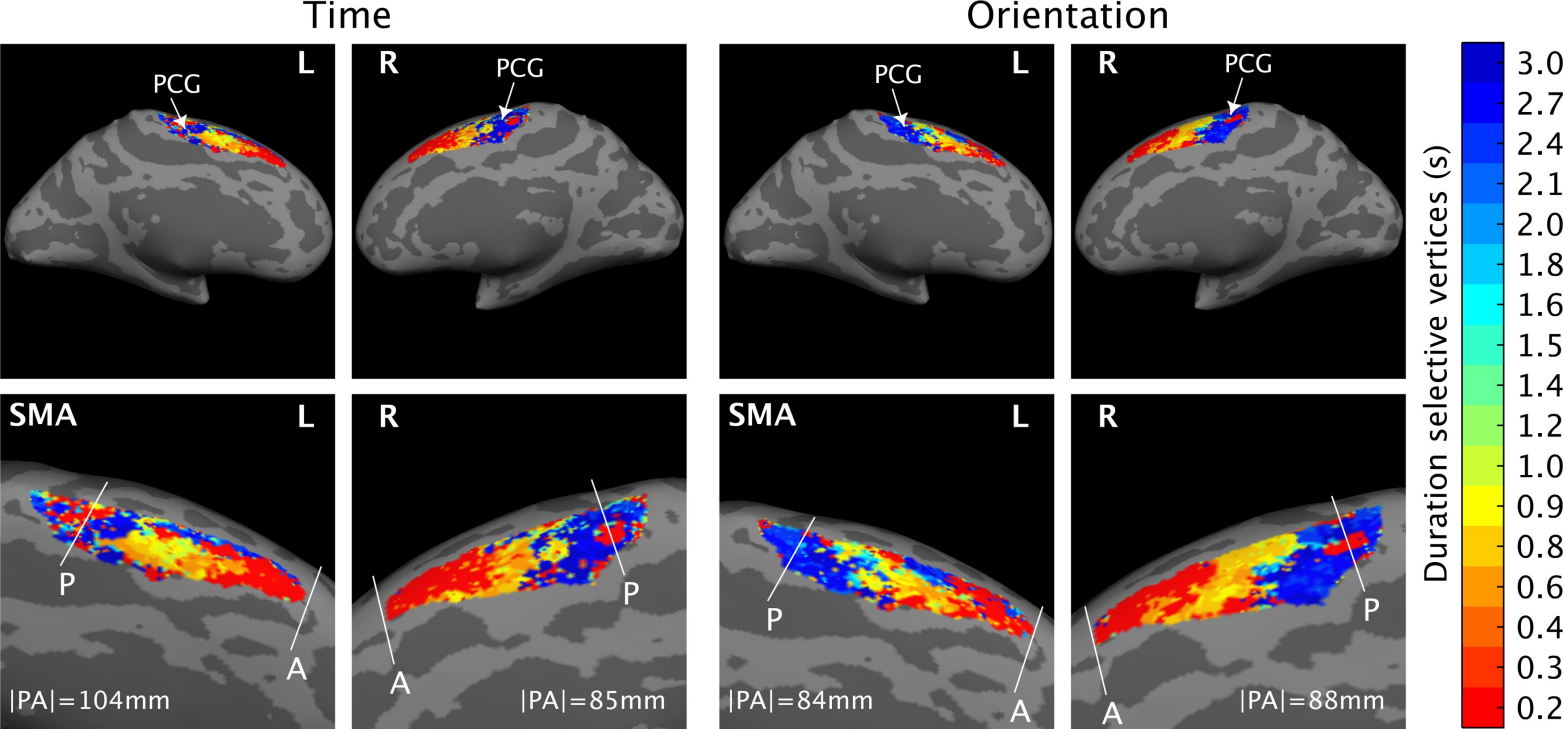
pRF group-level results of Exp 2. Here, we show the estimated μ parameter on the cortical surface (medial part of BA 6). Different colors represent vertices (i.e., voxels projected onto the cortical surface) selective to different duration ranges (i.e., vertices with different estimated μ). We show the results of the group (average of 10 subjects) for the 17 estimated μ. The μ are the 17 durations presented in the 10 different trial types (S1 duration was either a standard or a comparison duration). The color scale goes from red, i.e., shortest duration (0.2 s), to dark blue, i.e., longest duration (3 s). The white lines give an example of the map borders, as they were drawn to estimate the wRD in the individual subjects. For the wRD computation, borders were drawn in each individual subject in their native space. The size of the maps reported in the figure is the average size calculated across the 10 subjects when their maps were in a common Dartel space. On the left-hand side are time maps in time task; on the right-hand side are time maps in the orientation task. The data can be found in S2 Data. A, anterior; BA, Brodmann Area; L, left; P, posterior; PCG, precentral gyrus; pRF, population Receptive Field; R, right; SMA, Supplementary Motor Area; wRD, weighted relative distance.

Fig 6 (see S2 Data) shows the projection on the cortical surface (medial part of Brodmann Area 6 [BA 6]) of the estimated μ parameter at the group level. Different colors represent vertices (i.e., voxels projected onto the cortical surface) selective to different duration ranges (i.e., vertices with different estimated μ).

As indicated by the gradual change of color in brain activations shown in Fig 6, we found a topographic organization of duration-sensitive vertices in the left SMA replicating the results of Exp 1. In addition to the first experiment, here, we observed chronotopic maps for a broader range of durations, in both the left and the right hemisphere and for both the temporal and the orientation task (see leftward and rightward panels of Fig 6).

As in Exp 1, this progression was in the rostro–caudal direction within the SMA, with vertices sensitive to the shorter duration (vertices in warmer colors) located in the anterior and those sensitive to the longer duration (vertices in colder colors) in the posterior SMA.

In analogy with Exp 1, we looked at the spatial progression of chronomaps using three distinct indexes: wRD, PDs, and wCntrs (see Materials and methods for details). All of these indexes were computed for each individual subject. While wRD and PDs were estimated in the subjects’ native space, the wCntrs were estimated on data normalized to a common Dartel space. PDs and wCntrs were calculated at volumetric level, whereas wRD was calculated at surface level.

**Fig 7:**
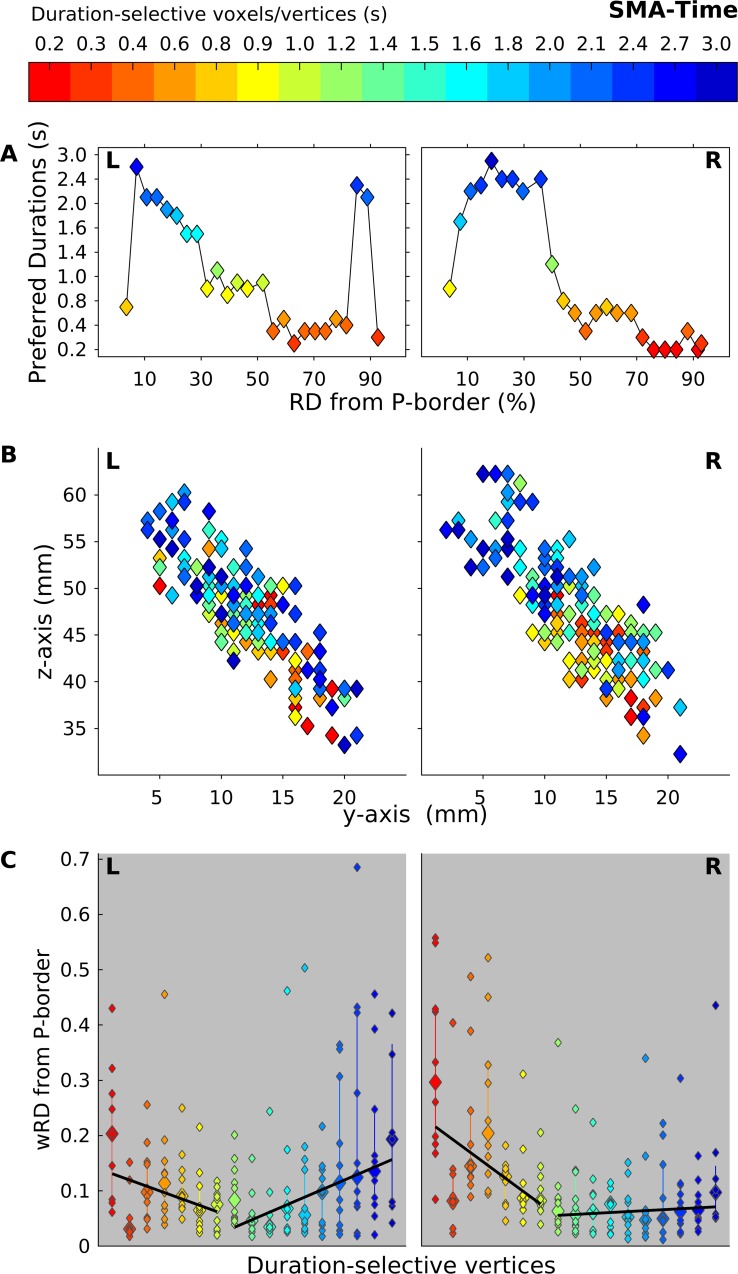
Spatial progression of L and R SMA chronomaps in Exp 2 during the time task. (A) Group average of PDs (y-axis) of voxels lying at different distances (x-axis RD) from the P border of the chronomaps. (B) 2D projection of wCntrs in the y-z plane for duration-selective voxels in SMA. wCntrs are color coded according to duration selectivity. The color scale goes from red (shortest duration 0.2 s) to dark blue (longest duration 3 s). Different colors indicate voxels with different duration selectivity; diamonds with the same color are the different subjects (*n* = 10). This value differs across duration conditions because not all subjects had the full range of duration-selective voxels. (C) For each duration-selective cluster of vertices, the full distribution of individual wRD (smaller diamonds) and their median value (biggest diamonds) are shown. A fitted slope based on these median values across subjects is also shown. The slope is calculated separately for durations below and above 1 s. The data can be found in S1 Data. L, left; P, posterior; PD, preferred duration; R, right; RD, relative distance; SMA, supplementary motor area; wCntr, weighted centroid; wRD, weighted relative distance.

**Fig 8:**
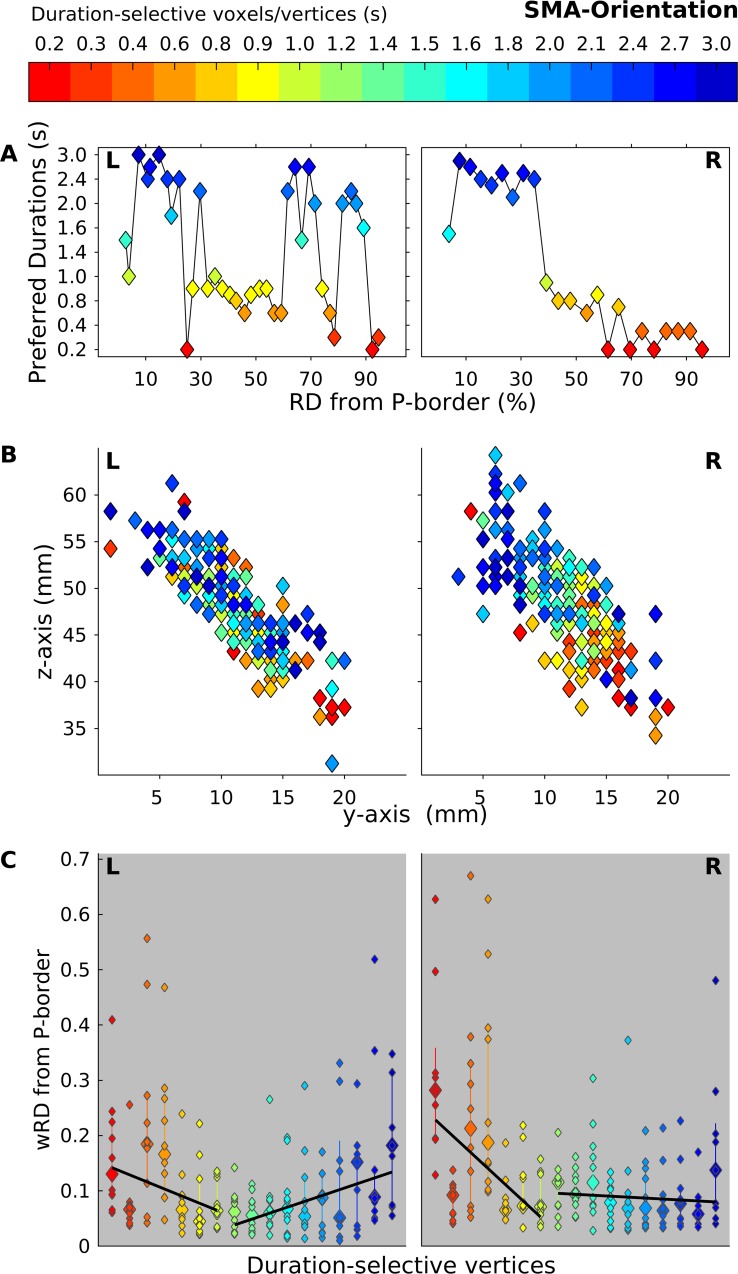
Spatial progression of L and R SMA chronomaps in Exp 2 during the orientation task. (A) Group average of PD (y-axis) of voxels lying at different distances (x-axis RD) from the P border of the chronomaps. (B) 2D projection of wCntrs in the y-z plane for duration-selective voxels in SMA. wCntrs are color coded according to duration selectivity. The color scale goes from red (shortest duration 0.2 s) to dark blue (longest duration 3 s). Different colors indicate voxels with different duration selectivity; diamonds with the same color are the different subjects (*n* = 10). This value differs across duration conditions because not all subjects had the full range of duration-selective voxels. (C) For each duration-selective cluster of vertices, the full distribution of individual wRD and their median value are shown. A fitted slope based on these median values across subjects is also shown. The slope is calculated separately for durations below and above 1 s. The data can be found in S1 Data. L, left; P, posterior; PD, preferred duration; R, right; RD, relative distance; SMA, supplementary motor area; wCntr, weighted centroid; wRD, weighted relative distance.

The PDs and the centroids show results that are consistent with what was observed for the maps at the group level. For the PDs (Fig 7A and Fig 8A for time and orientation task, respectively; see also S1 Data), we found for both tasks and both hemispheres that the voxels lying closer to the posterior border of the chronomap preferred the longer durations, whereas those lying furthest preferred the shortest duration. For the centroids, in both hemispheres and tasks, in the majority of the tested subjects, the clusters of voxels selective to the shorter durations had centroids located more anteriorly (see the y-axis, diamonds in warmer color) with respect to the voxels responsive to the longer durations (diamonds in colder color).

Although in a visual inspection (see Fig 6, Fig 7A and 7B, and Fig 8A and 8B) of the group-level results chronotopic maps seemed to include the whole range of durations tested, the analysis on the wRD revealed an interesting dissociation between sub- and suprasecond durations in the spatial progression of the maps (see Fig 7C and Fig 8C). An analysis on individual slopes based on wRD and calculated for sub- and suprasecond durations (Wilcoxon sum rank test) showed the presence of a significant difference (i.e., negative slope versus slope equal to zero) for subsecond durations in the temporal and orientation tasks for both the left and right hemispheres (*P* < 0.001). For the suprasecond durations, the slope was either flat or positive (left SMA in time and orientation tasks *P* < 0.001). No statistically significant difference was observed between tasks (neither for sub- or suprasecond durations *P* > 0.6). This result shows that the spatial progression in SMA chronomaps is clear for subsecond duration in bilateral SMA and for both the time and the orientation tasks.

For individual maps, see S8–S11 Figs and S4 Data. For left and right SMA in the temporal task, see S8 Fig and S9 Fig. For the left and right SMA in the orientation task, see S10 Fig and S11 Fig. For a better appreciation of individual slopes, see also S12 Fig and S3 Data.

Within the IPS, we did not find a clear topography, neither at the group nor at the single subject level (see S13 Fig and S4 Data).

**Fig 9:**
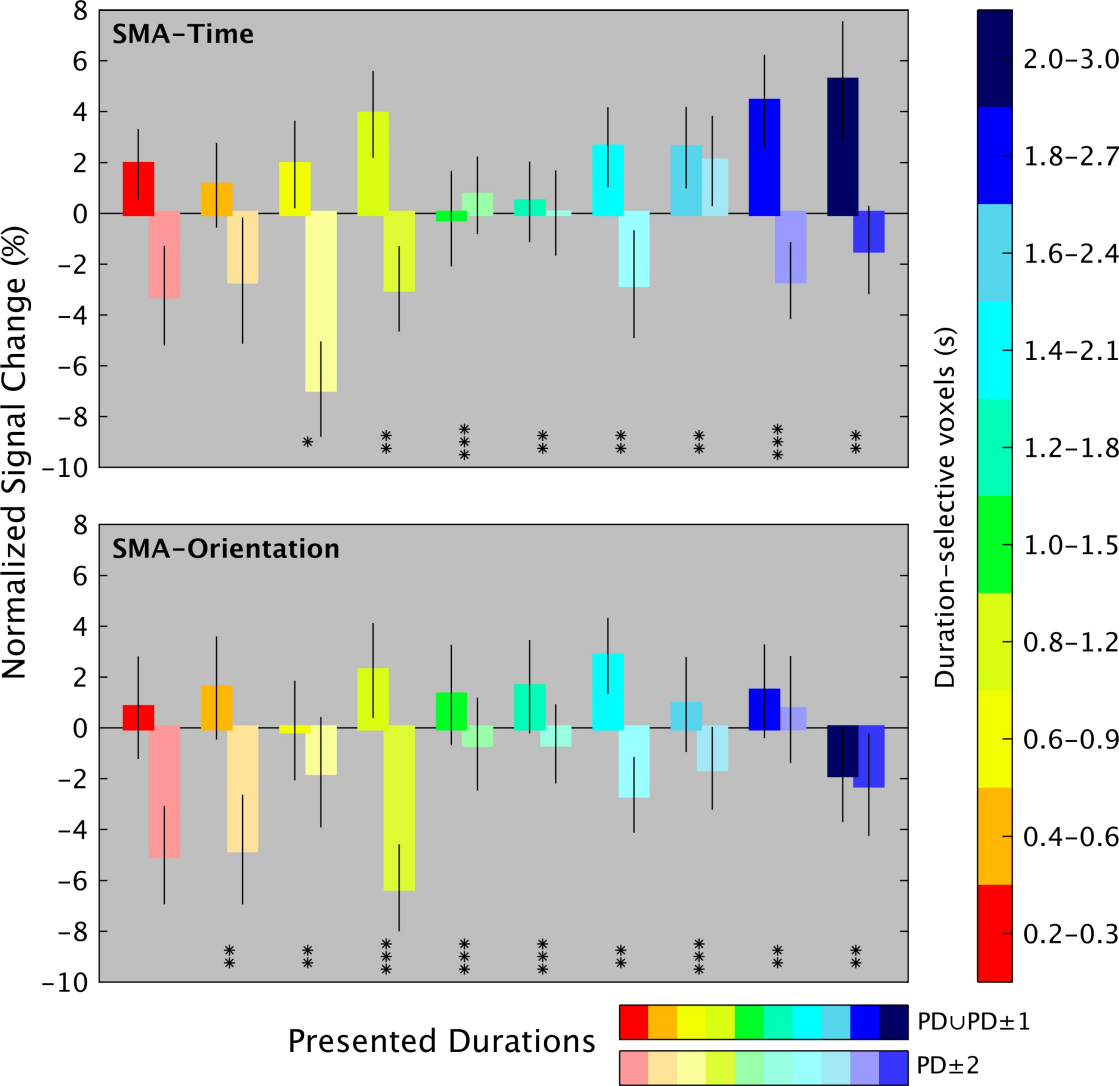
Duration tuning of Exp 2. Group average of normalized BOLD responses of duration-selective voxels (colored bars, y-axis) for PD and neighboring (PD ± 1) durations (PD ∪ PD ± 1, bars with darker shades), as opposed to distant nonpreferred durations (PD ± 2, bars with lighter shades). Asterisks indicate statistically significant difference at a Wilcoxon rank sum test between PD ∪ PD ± 1 and PD ± 2 at *P < 0.01, **P < 0.005, and ***P < 0.001. Given the limited number of trials for each of the 17 presented durations for this plot, we grouped the duration-selective voxels according to the 10 different trial types. On the x-axis are the 17 presented durations grouped in 10 different duration ranges. The BOLD signal in duration-selective voxels is aligned to the presentation timings of the different duration ranges (i.e., second volume after S1 offset). The data can be found in S1 Data. BOLD, blood oxygenation level-dependent; PD, preferred duration; S1, first stimulus.

To examine the response tuning of duration-sensitive voxels in the second experiment, we looked at the variation of the hemodynamic response as a function of the presented duration, i.e., preferred versus nonpreferred durations. Fig 9 (see S1 Data) shows the normalized hemodynamic response of SMA duration-selective voxels to PD and neighboring durations (PD ± 1, see darker shades), as opposed to the response to distant durations (PD ± 2, see lighter shades). Given the limited number of repetitions for each of the 17 presented durations, to plot the signal change, we grouped the durations according to the 10 different trial types (i.e., 10 pairs of durations). The normalized BOLD response is plotted for both time (upper panel) and orientation tasks (lower panel). The bar plot shows that for the majority of duration-selective voxels, activity was enhanced for preferred (as expected) and neighboring durations and suppressed for more distant durations (see Fig 9). Since there was no difference in the tuning analysis of left and right hemispheres, the plot shows the average tuning of left and right SMA. As a complementary check of the duration tuning, we looked at the normalized signal change of the shortest- and the longest-duration–selective clusters of voxels over the time of a cycle (i.e., 44 seconds/22 TRs; see S14 Fig and S3 Data). For both clusters, the hemodynamic response peaked and dropped at the appropriate times in a cycle, i.e., early in the cycle for the shortest-duration–selective cluster and later in the cycle for the longest-duration–selective cluster. The same pattern, although less clear, was present for the orientation task.

**Fig 10:**
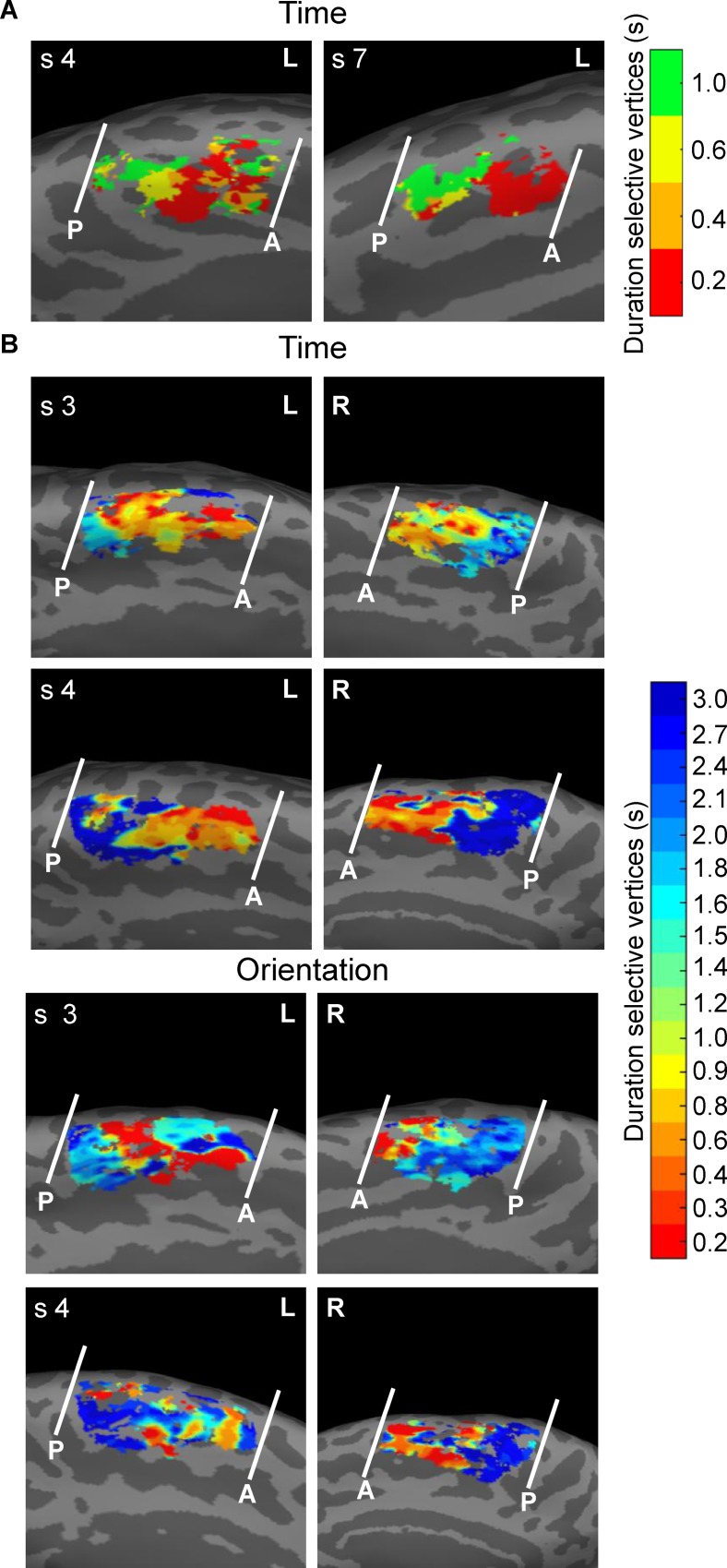
**fMRI results, individual data of Exp 1 (A) and Exp 2 (B).** (A) For two subjects of Exp 1 we show the left SMA chronomap with the A and P borders. Individual maps were obtained using a winner-take-all procedure based on statistical t-maps (T > 3.13). We computed four different t-maps for each of the four S1 durations (*P*_FWE_ < 0.05, cluster level corrected for multiple comparisons across the whole brain). For the maps of whole sample (*N* = 11) of subjects, see S2 Fig. (B) For two subjects of Exp 2, we show the left and the right SMA chronomap with the A and P borders in the time (leftward) and in the orientation (rightward) tasks. The maps were the results of a pRF analysis. Here, we show the projection on the cortical surface (medial part of BA 6) of the μ parameter. The μ are the 17 durations presented (S1 when is either the standard or the comparison duration) in the 10 different trial types. Different colors represent vertices selective to different duration ranges (i.e., vertices with different estimated μ). For the maps of whole sample (*N* = 10) of subjects, see S6–S9 Figs. The data can be found in S2 Data. A, anterior; BA, Brodmann Area; fMRI, functional magnetic resonance imaging; FWE, familywise error; P, posterior; pRF, population Receptive Field; S1, first stimulus; SMA, supplementary motor area.

At this point, it is worth emphasizing here that in both experiments, we observed a certain degree of variability in SMA chronomaps across subjects. Fig 10 (see S2 Data) shows for Exp 1 (panel A) and Exp 2 (panel B) the SMA chronomaps in two “ideal” subjects, i.e., subjects with an anterior-short to posterior-long spatial progression. This progression was present in seven out of 11 subjects in Exp 1 (left SMA) and in nine out of 10 subjects in Exp 2 (bilateral SMA; see S2 Fig and S8–S11 Figs for the SMA maps of all subjects).

To investigate whether the individual variability in the spatial progression of the SMA chronomaps was linked to the behavioral performance in the temporal task, we correlated (using Kendal's tau correlation coefficient) the slope of the wRD measured in SMA (i.e., left SMA for Exp 1 and bilateral SMA for Exp 2) with two behavioral indexes of temporal performance: accuracy and coefficient of variation (i.e., CV = standard deviation/duration). The results (see S15 and S16 Figs and S3 Data) showed that the better the spatial progression of the map (i.e., steeper negative slope), the more accurate and less variable the subject’s performance is. While the result is very robust for Exp 1 (for accuracy: tau = −0.64, *P* < 0.05; for CV: tau = 0.64, *P* < 0.05; S15 Fig), it is not statistically significant for Exp 2 (accuracy SMA left: tau = −0.25, SMA right: tau = 0.15; CV SMA left: tau = 0.15, SMA right: tau = −0.15; S16 Fig).

**Fig 11:**
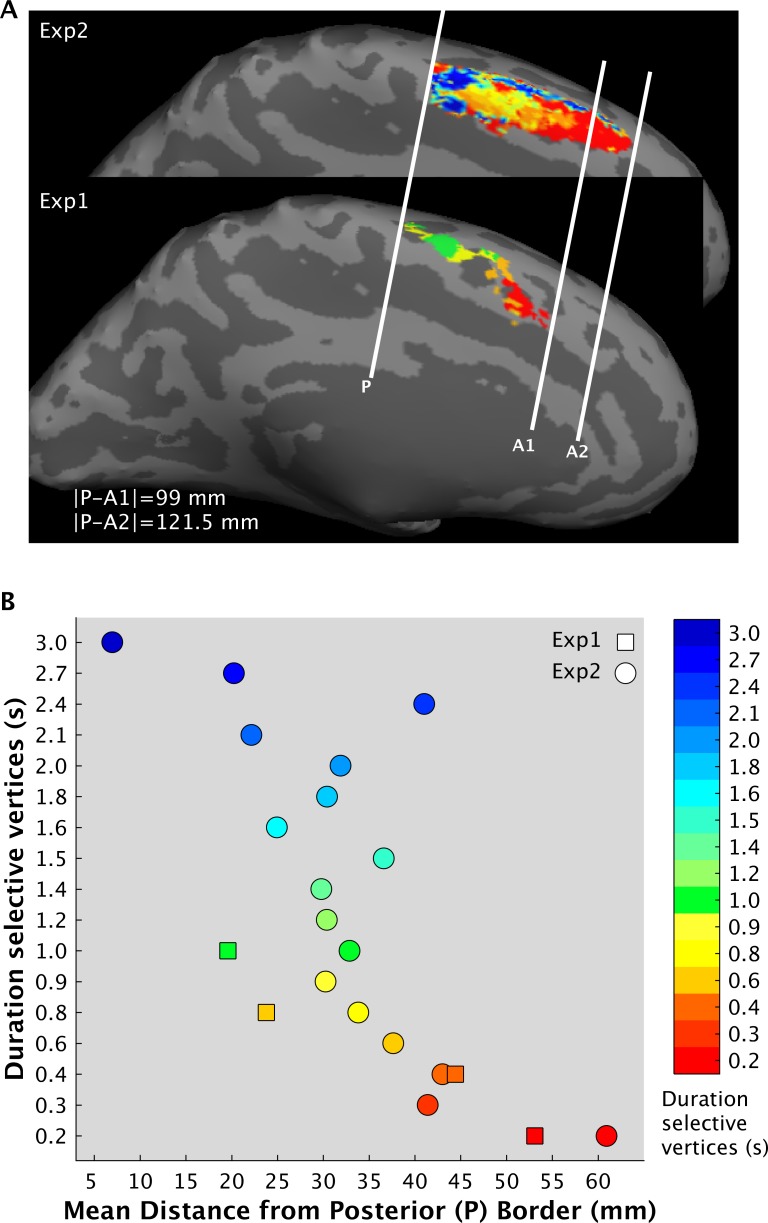
fMRI results, comparison between the chronotopic maps of the two experiments. (A) Chronotopic maps in the left SMA in the two experiments; group-level maps are superimposed on an inflated Dartel template computed on the high-resolution anatomical images of the 21 subjects. White lines are the borders of the maps. The posterior border lies on the most posterior part of the PCG. (B) For each experiment (squares are Exp 1 and circles Exp 2) and for each duration-selective clusters of vertices, we estimated the centroids and then calculated their absolute distance from the posterior border. The data can be found in S1 Data. fMRI, functional magnetic resonance imaging; PCG, precental gyrus; SMA, supplementary motor area.

Finally, in order to check whether SMA chronomaps represent duration in a relative or absolute fashion, we compared the spatial distribution of the maps in the two experiments. Fig 11 shows the SMA chronomaps of the left hemisphere in the two experiments (data are now in a common space, i.e., Dartel template computed on the high-resolution anatomical images of the 21 subjects). The figure shows that in Exp 2, for which we used a wider range of durations, the chronomap has a bigger size (121 versus 99 mm), and the duration-selective clusters that are common in the two experiments are anteriorly shifted (see Fig 11B and S1 Data; 0.2 s = +7.8 mm, 0.4s = −1.4 mm, 0.6s = +10 mm, 1s = +13 mm). As a consequence of this shift, only a small proportion of vertices had the same selectivity across the two experiments (on average is the 5% of total number of voxels selective for a given duration). This last result suggests that SMA chronomaps represent durations in a relative rather than an absolute manner.

We also compared the size of the different duration-selective clusters in the two experiments (see S17A–S17C Fig and S3 Data). In Exp 1, the four clusters were equally represented (Wilcoxon rank sum test *P* > 0.23). In Exp 2, for both tasks, the size of the 17 duration-selective clusters was unequal, i.e., the shortest (0.2 s) and the longest (3 s) durations were more numerous than the intermediate ones (from 0.8 to 2 s, for all durations, *P* < 0.05; ANOVA task x durations; effect of durations F = 12.69, *P* < 0.01).

## Discussion

To summarize, we showed with two independent data sets, paradigms, and methods of data analysis that different portions of SMA responded preferentially to different durations. Duration selectivity had a clear topographical organization in the rostro–caudal direction for short and long durations, respectively. Although chronotopic maps were observed across a wide range of durations (from 0.2 to 3 s), the topographical arrangement of duration-selective vertices was better for subsecond durations. Chronotopic maps were observed not only at the group level but also, with a certain degree of variability, at the single-subject level. The individual variability of the maps seemed to be linked to participants’ temporal performance, i.e., the more accurate and precise the performance, the better the spatial progression of the map. Chronotopic maps were also task independent; maps were indeed found when time was available, but it was task irrelevant. At the tuning level, we found that the hemodynamic response in duration-selective voxels was enhanced by neighboring durations and suppressed by durations far from the preferred one. Finally, SMA chronomaps represented durations in a relative rather than absolute manner; the size of the map and the tuning of the duration-selective clusters changed with changes of the duration range tested.

Neuronal tuning and topography are encoding mechanisms widely used in neurons to represent sensory and motor information [13][19] and even more abstract features like quantities [14]. This topographic organization, which can have a certain degree of redundancy in the brain (e.g., the multiple visual spatial maps), is thought to have a computational benefit, e.g., improving the efficiency of neural communication [20]. In the context of temporal perception, given the potentially unlimited duration span, a mechanism of this sort can be beneficial when multiple durations have to be simultaneously represented and recognized. The observation that SMA chronomaps adjust their tuning to the duration range at hand, although is not yet conclusive and needs further testing, is nonetheless a result in line with this hypothesis of efficient coding of multiple durations.

Chronomaps have been identified for the first time in human SMA. Duration-selective cells have been previously reported in monkeys’ medial premotor cortex [3][4]. The present study extends this representational format to humans and shows that duration-selective units in this region are topographically organized along the anterior-to-posterior axis. Moreover, while the presence of duration-selective units in monkeys’ premotor cortex was exclusively associated with motor-timing behavior, our study shows the presence in human premotor cortex of duration-selective mechanisms in a purely temporal perceptual task.

In humans, duration-selective mechanisms have been recently suggested by an fMRI study showing duration adaptation effects in the activity of the inferior parietal lobule (i.e., the supramarginal gyrus) [11]. Activity in this region is suppressed when consecutive stimuli have the same duration. Our data support this finding and show the presence of duration-selective mechanisms in a closer location, i.e., the IPS, although in the left rather than the right hemisphere. However, our data go beyond this previous finding by showing a) the existence of duration-selective activity for a wider range of durations, b) duration selectivity not only in the IPS but also in the SMA, and, c) most importantly, we showed that only activity in the SMA is topographically organized in a way that neuronal units selective to similar durations occupy contiguous portions of the cortical surface so as to form chronomaps.

Compared to our study, Hayashi and colleagues [11] failed to find adaptation effects in SMA. The lack of this effect can be explained by a fundamental methodological difference between the two studies. Hayashi and colleagues (2015) focused their fMRI analysis on the S2 of a pair, a stimulus immediately followed by a motor response. It could be therefore possible that in SMA, the repetition-suppression signal was masked by the ramping signal of the motor preparation. Our study instead focused on the brain response associated with the S1 of the pair; a response that is well dissociated from a motor preparation–related signal. We therefore believe that our method of analysis, being more focused on the encoding stage of the task, was better suited to reveal the contribution of SMA.

The presence of topography in SMA but not in IPS may indicate that duration selectivity in different brain regions (IPS and SMA) serves different purposes along the process leading to duration judgments. Our hypothesis is that duration-selective activations in the premotor cortex may reflect an active reconstruction of temporal signals coming from different regions of the brain (e.g., visual or parietal areas) [21][2][22]. One can think of chronomaps in SMA as a temporal read-out, a later stage of duration encoding in which duration information becomes finally available and decision-making takes place. The IPS duration selectivity, which lacks a clear topography [11], may represent an intermediate stage in which duration signals coming from low-level sensory regions are automatically organized. A support to this hypothesis comes from the observation that the perturbation of right supramarginal gyrus activity via Transcranial Magnetic Stimulation (TMS) affects time representations in the SMA [22].

Two further observations are in line with the idea that chronotopic maps do not represent a low-level stage of temporal processing: the anatomical location of the maps and the link between spatial progression of the maps and both accuracy and precision of temporal judgments. Chronomaps were mainly observed in SMA and neither in the parietal nor in sensory regions. SMA has been implicated in a variety of timing tasks [16][23][24] with a range of durations spanning from a few hundreds of milliseconds to a few seconds [25][26] and with stimuli from different sensory modalities [27][28][29]. It is therefore likely that this area constitutes an “amodal” and “high-level” core of a timing network in which duration is represented in an abstract form independent of specific sensory modality or motor behavior. The correlation between spatial progression of the maps and performance is in line with a previous fMRI study showing a correlation between the hemodynamic response in SMA and the perceived duration (i.e., greater BOLD response led to time overestimation) [30].

The role of SMA as “amodal” and “high-level” time read-out is also in line with a growing body of evidence showing a tight link between motor planning and visual time perception [31][32][33]. One of those studies [32], for example, shows that the preparation of isometric contractions and real movements of the hand biases the time perception of visual events, i.e., time perception expands for hand movements that point away from the body and compresses for movements toward the body. SMA, for its role in both time perception and action planning, likely plays a key role in this temporal sensory-motor integration.

At this point, we would like to emphasize here that even if chronomaps are found in SMA and duration tuning is observed in both SMA and IPS, we still believe that time perception is the result of the activity of a wide network of brain regions, including sensory [21,34], motor [30,35], and associative cortices [15,36].

SMA chronomaps were also observed during the orientation discrimination task when the temporal dimension of the stimuli was task irrelevant. This last result may at first glance appear contradictory to the link between quality of the maps and behavioral performance. However, we would like to point out here that the orientation discrimination task required participants to attend the stimulus orientation’s changes over time. It may therefore be possible that while appreciating stimulus orientation, participants also attended to the temporal dimension of the stimuli. That could be indeed the reason why we observed maps in the orientation task. More research would be necessary to better specify the role of attention in modulating chronomaps’ activity. In particular, it would be interesting to check whether the attention payed to the stimulus’s duration, even when task irrelevant, depends on the fact that participants know that in each experimental session, they are going to judge both the time and the orientation of the stimuli. In other words, it would be important to see whether SMA chronomaps are present when participants are exclusively engaged in a nontemporal task.

Chronomaps covered the whole length of SMA, from SMA-proper to pre-SMA. The spatial progression was in the rostro–caudal direction, with clusters preferring the shorter duration located anteriorly compared to those preferring longer durations.

This progression does not fit any of the known motor or cognitive functional specializations of SMA. In the time domain, pre-SMA is engaged preferentially by perceptual timing tasks and by timing of sequences, whereas SMA-proper is preferentially recruited by motor-timing tasks and by timing of single events [37–39]. In the motor domain, pre-SMA is often linked with self-initiated movements, with response inhibition or task switching, whereas SMA-proper with stimulus driven movements and motor preparation [40]. A somatotopically arranged body map is also present in SMA. Stimulation studies conducted in nonhuman primates show that movements of the hind limb are evoked from caudal sites, whereas forelimbs and oro-facial movements are evoked from more rostral sites [41][42]. Linking the spatial progression of the chronomaps with any of the above-mentioned distinctions is rather difficult. More research is needed to clarify the relationship between chronomaps and the well-known functional properties of SMA in both the time and the motor domain. In addition, further studies are warranted for clarifying whether chronomaps reported here are linked to a specific sensory modality or a specific temporal task or whether they represent perceived or physical duration or if maps can be distorted by pharmacological manipulations or learning.

An interesting observation came from Exp 2 in which a wider range of durations was tested. By quantifying the spatial progression of the maps, we found that the rostro–caudal spatial progression worked better for subsecond rather than suprasecond durations. This result is in line with a few psychophysical [43,44] and neuroimaging observations [26,45], suggesting the existence of distinct mechanisms and neuronal substrates for the processing of durations above and below the second.

Duration-selective units were responsive to neighboring durations and exhibited the strongest suppression to durations distant from the preferred one. This seems to suggest a Gaussian-like type of response profile, in which neuronal units tuned to similar durations have overlapping tuning curves. This tuning profile is also in line with the behavioral effects obtained with duration adaptation paradigms in which an optimal proximity between “adaptor” and test duration leads to stronger repulsive effects [9]. In analogy with spatial vision or audition (e.g., visual orientation [13] or auditory pitch [46]), the tuning profiles observed here may serve the function of enhancing the discriminability of durations by suppressing the activity for different durations.

In summary, we found a topographic representation of time in the human SMA, an area that has been previously identified as a “time” region. Our findings of chronomaps clarify the nature of duration information represented there and, most importantly, indicate duration tuning and topography as possible mechanisms for duration read-out.

## Materials and methods

### Subjects

We tested a total of 21 healthy volunteers, 11 in Exp 1 (5 females, mean age 23.7 years, SD 4.3 years) and 10 in Exp 2 (9 females, mean age 27.7 years, SD 5.1 years) with normal or corrected-to-normal vision. All volunteers gave written informed consent to participate in this study, the procedures of which were approved by the ethics committee of the Faculty of Biology and Medicine at the University Hospital of Lausanne (protocol number 92/2012) in accordance with the Declaration of Helsinki.

### Stimuli and procedure

In Exp 1, we used a temporal discrimination task of visual durations. Visual stimuli were sinusoidal Gabor patches (100% contrast, spatial frequency of 1.9 cycles/degree, Gaussian envelope with standard deviation of 2.2 degrees, diameter of approximately 9 degrees) with a circular hole (diameter 0.6 degrees at the center of the Gabor) displayed at the center of the screen around a central fixation spot (a black disk 0.5 degrees of diameter at a viewing distance of 90 cm) on a gray background. In each trial, two Gabor patches (S1 and S2) were sequentially presented with a variable interstimulus interval ranging between 4 and 5.2 s in 0.08 s steps. The two stimuli were followed by a response cue, i.e., a red fixation spot of 2-s duration (see Fig 1A). S1 and S2 varied in orientation and duration, although only duration was task relevant. The duration of S1 could be 0.2, 0.4, 0.6, and 1 s and its orientation 36, 72, 108, and 144 degrees. S2 could be either shorter or longer in duration than S1. The duration of S2 was longer or shorter by a constant Weber ratio of 0.4 (e.g., if S1 was 0.2 s, S2 was either 1.6 or 3.6 s), whereas the orientation of S2 was a value randomly chosen from the four possible orientations used for S1 (i.e., 36, 72, 108, or 144 degrees). The combination of duration and orientation lead to 16 different types of S1 stimuli. Each stimulus type for S1 was presented only once in each fMRI run.

Participants were asked to judge whether the duration of S2 was shorter or longer than S1. Participants made their responses by pressing one of two buttons on a response pad. They used their right index finger to express the choice “S2 shorter than S1” and their right middle finger for the “S2 longer than S1” responses. Participants were instructed to be as accurate as possible (no emphasis was put on reaction times) and to fixate at the center of the screen while performing the duration discrimination task. They were also requested to ignore the orientation changes of the stimulus and to not use counting strategies to estimate duration.

Each fMRI run contained 16 trials, and the total duration of each run was 3 min and 51 s. We collected 18 fMRI runs in two separate sessions (9 runs per session). The second session was performed 1–3 days after the first session. The data of this first experiment are partially shared with another study [47].

In Exp 2, two tasks were used: a temporal discrimination and an orientation discrimination task. The stimuli and the task structure were identical in the two tasks; the only difference was that the stimulus feature participants were asked to attend (duration versus orientation). The stimulus was a sine wave grating (size = 400 by 400 pixels, 8.01 degree of visual angle at viewing distance of 90 cm; spatial frequency was 0.05 cycle/pixel), drifting at a speed of 1 cycle per second and displayed at varying angular orientations. Within a trial, the sequence of orientation changes was fixed and was always leftward first, rightward second, and vertical last (Fig 1C). Within the two “main orientations” (leftward–rightward), the grating continuously changed its orientation at a rate of 5 Hz (an orientation change each 0.2 s), and the range of changes was between 30° and 45°. The amount of time the grating maintained its “main” orientation defined a temporal interval. During the temporal discrimination task, participants judged which of the two “main orientations” (leftward or rightward) was maintained for a longer time. In the orientation discrimination task, participants judged which of the two “main orientations” underwent the biggest angular change. In this manner, the physical stimuli were identical, and the amount of attention paid to them was equated across tasks. The only difference between tasks was the instruction given to the participants (attend to duration versus attend to orientation changes). The vertical orientation signaled the time to make the response (by pressing one of two response keys on a keypad), and it was also the intertrial interval. The duration of the vertical orientation was kept constant (1.37 s), whereas the duration of the two “main orientations” varied.

For each trial, there was always a standard (T) and a comparison duration (T + ΔT). The duration of the comparison was a constant proportion of the standard (i.e., 50% of the standard, Weber ratio was equal to 0.5). The presentation order of standard and comparison (i.e., standard first, comparison second or vice versa) was randomized and counterbalanced across trials. Half of the time, S1 was a standard, and the other half, it was a comparison duration. We used 10 different standard durations, ranging from 0.2 to 2 s in steps of 0.2 s, one for each trial. The full combination of standards and comparisons resulted in the following 10 pairs of durations: 1: 0.2–0.3 s, 2: 0.4–0.6 s, 3: 0.6–0.9 s, 4: 0.8–1.2 s, 5: 1.0–1.5 s, 6: 1.2–1.8 s, 7: 1.4–2.1 s, 8: 1.6–2.4 s, 9: 1.8–2.7 s, and 10: 2.0–3.0 s.

The overlap between duration pairs was a consequence of choosing comparison durations that were 50% of the standard duration. We chose such a relatively high Weber fraction to make sure that the difference between standard and comparison duration was clearly perceived. Since we analyzed the brain response at the offset of S1 duration and since S1 could be either standard or comparison, we were able to capture the signal correlated to 17 different durations independently from the pairs they belonged to.

While the grating was displayed for a standard and a comparison duration, its angular orientation changed at a rate of 5 Hz. The angular change was one of 12 pseudo-randomly chosen values ranging from 30° to 45° (in logarithmic steps, base 10). It is worth emphasizing here that since the orientation changes were chosen pseudo-randomly, sometimes the same orientation could be displayed more than once (maximum number of allowed repetitions of the same orientation was three). Therefore, the number of orientation changes was not entirely predictive of the duration of the stimulus.

The differences between rightward and leftward orientation could be 5°, 7°, 9°, or 11°. We chose these different values based on the results of a purely behavioral pilot study for which we tested both temporal and orientation discrimination tasks. The angular differences chosen were those leading to discrimination accuracy similar to the temporal task.

Both tasks were structured in “ascending” and “descending” cycles. Each cycle comprised 10 trials and lasted 44 s. “Ascending” cycles started with the shortest duration pair (i.e., 0.2–0.3 s, first trial) and ended with the longest pair (i.e., 2–3 s, the tenth trial). In descending cycles, it was the reverse (i.e., the first trial had the longest and the tenth the shortest pair). The time interval between cycles was 2.03 s; during this interval, the grating was in vertical orientation. In both tasks, subjects were responding using either the index or the middle finger of their right hand. In each fMRI run, there were 10 cycles. There were two separate runs for “descending” and “ascending” cycles (one run each) and two separate runs for the temporal and the orientation discrimination tasks. The order of the tasks was counterbalanced across subjects (i.e., half of the participants performed the two time runs first; the other half performed the two orientation runs first). Each participant thus performed a total of four fMRI runs (220 fMRI volumes each).

### Behavioral data analysis

In Exp 1, for each participant, we took the percentage of performance accuracy for the four different S1 durations, and we entered these values in a one-way repeated measures ANOVA.

In Exp 2, for each participant, we took the percentage of performance accuracy for the 10 different duration pairs in the two tasks and submitted them to a task (time, orientation) × durations (10 durations pairs) within subject ANOVA.

For both experiments, the alpha level was set to 0.05. As posthoc test, we used the Bonferroni test.

### MRI acquisition and analyses

#### MRI acquisition

The mapping of the selectivity of the neural responses necessitated high spatial resolution of the functional data. The increased signal-to-noise ratio and available BOLD associated with ultrahigh magnetic field systems (>3 T) allowed the use of smaller voxel sizes in fMRI [48]. In addition, the spatial specificity of the BOLD signal is improved because the signal strength of venous blood is reduced due to a shortened relaxation time, restricting activation signals to cortical gray matter [48]. Therefore, we employed high-resolution, 7T fMRI for the functional maps.

In both experiments, BOLD functional imaging was performed using an actively shielded, head-only 7T MRI scanner (Siemens, Germany), equipped with a head gradient insert (AC84, 80 mT/m max gradient strength; 350 mT/m/s slew rate) and 32-channel receive coil with a tight transmit sleeve (Nova Medical, Massachusetts, United States of America).

In Exp 1, time course series of 169 volumes were acquired for each run, using the 3D-EPI-CAIPI sequence [49]. The spatial resolution was 2.0 mm isotropic, the volume acquisition time was 1,368 ms, the flip angle was 14 degrees, the repetition time (TR) 57 ms and the echo time (TE) 26 ms, and the bandwidth 2,774 Hz/Px. The matrix size was 106 x 88 x 72, resulting in a field of view of 210 (AP) x 175 (RL) x 144 (FH) mm. An undersampling factor 3 and CAIPIRINHA shift 1 were used. Slices were oriented transversally with the phase-encoding direction left–right. For the GRAPPA reconstruction, 42 x 45 reference lines were acquired. For each individual, a total of 3,042 volumes (169 volumes per run, 18 runs) were analyzed.

High-resolution whole-brain MR images were also obtained using the MP2RAGE (magnetization prepared rapid gradient echo) pulse sequence optimized for 7T [50] (voxel size = 1.0 x 1.0 x 1.0 mm, matrix size 256 x 256 x 176, TI_1_/TI_2_ = 750/2,350 ms, α_1_/α_2_ = 4/5 degrees, TR_MP2RAGE_/TR/TE = 5,500/6.5/2.84 ms).

In Exp 2, fMRI data were acquired with a continuous EPI pulse sequence with sinusoidal read-out (1.5 × 1.5 mm in-plane resolution, slice thickness = 1.5 mm, TR = 2,000 ms, TE = 25 ms, flip angle = 47°, slice gap = 1.57 mm, matrix size = 148 × 148, field of view 222 × 222 mm, 40 oblique slices covering most of occipital, parietal, and premotor regions). In each fMRI run, we acquired 220 fMRI volumes. A T1-weighted high-resolution 3D anatomical image (resolution = 1 × 1 × 1 mm, TR = 5,500 ms, TE = 2.84 ms, slice gap = 1 mm, matrix size = 256 × 240, field of view = 256 × 240) was acquired for each subject using the MP2RAGE pulse sequence. For each participant, an additional whole-brain EPI image (a single volume with 80 slices and TR = 4,000 ms and otherwise identical parameters to the functional data) was acquired in order to aid the coregistration between the EPI images and the individual MP2RAGE. The EPI sequence used in Exp 2 did not allow whole-brain coverage. Based on the results of Exp 1, we chose to place the 6-cm thick imaging slab so as to cover the occipital, parietal, and premotor cortices.

#### fMRI preprocessing

For both experiments, functional imaging data were preprocessed using the statistical parametric mapping toolbox (SPM12, Wellcome Department of Imaging Neuroscience, University College London). In Exp 1, the EPI volumes acquired in each session were realigned to the mean of the session and then coregistered to the T1-weighted image acquired in the same session. In order to perform group level analysis (see Fig 2), the realigned and coregistered images were then normalized to the averaged DARTEL template (diffeomorphic anatomical registration through exponentiated lie algebra [51]) and smoothed with a 2-mm full-width at half-maximum Gaussian kernel. To perform surface-based analysis, data were kept in the subject’s space, i.e., after realignment and coregistration to the T1-weighted image, data were then directly smoothed with a 2-mm full-width at half-maximum Gaussian kernel (Fig 3A and Fig 4).

In Exp 2, the EPI volumes acquired in each session were slice time corrected, realigned to the mean of the session, and coregistered first to the whole-brain EPI image and subsequently to the T1-weighted image acquired in the same session. Since the sequence used for Exp 1 was a 3D-EPI-CAIPI (i.e., the whole k-space was acquired at once, with no time lags), only in Exp 2 data were slice time corrected. In order to performed volumetric analyses and to visualize the group-level pRF results, a DARTEL temple was also created for Exp 2.

#### GLM analysis

Exp 1 data were analyzed using a GLM approach. The fMRI time series were first analyzed in each single subject. Each single subject model included 18 runs/session with six event types in each session. These comprised the four different S1 durations (each event was time locked to the offset of S1), a fifth event time locked to the onset of S2 (comparison duration), and a sixth event time locked to the onset of the participants’ response. The duration of the events was set to zero.

All events were convolved to the canonical HRF. We used event offset as onset of the GLM model because it was the moment when the duration of a stimulus became available to participants. Moreover, by modeling event offset, we minimized the contamination arising from the temporal integration of visual responses.

The linear models included also the motion correction parameters as effects of no interest. The data were high-pass filtered (cutoff frequency = 0.0083 Hz). In order to see brain activity correlated to the different S1, for each subject, we estimated four contrasts, one for each S1. These contrasts also averaged parameter estimates across the 18 runs.

In order to test the existence of chronomaps in the group, the four contrast images estimated in each subject were then entered into a second-level ANOVA for which we performed again four different contrasts (one for each S1 duration). The statistical threshold was set to *P* < 0.05 FWE cluster-level corrected for multiple comparisons across the entire brain volume (cluster size estimated at a voxel level threshold *P* uncorrected = 0.001).

Correction for nonsphericity [52] was used to account for possible differences in error variance across conditions and any non-independent error terms for the repeated measures.

To appreciate the existence of chronomaps, the four t-maps, obtained either at single subject or at group level, were then used to classify the voxels according to their preference to one of the four different duration ranges. Voxels were classified according to a winner-take-all rule, e.g., voxels with the greatest T value (threshold was set to T > 3.13) for the shortest duration range (0.2 s) were classified as responsive to that duration range and labeled with number 1. We created four different labels, and each label was associated with a specific color for visualization purposes.

#### pRF analysis

Data from Exp 2 were analyzed using the pRF method. The pRF analysis was performed with the SamSrf toolbox for pRF mapping (https://figshare.com/articles/SamSrf_toolbox_for_pRF_mapping/1344765/22).

This toolbox implements a method of analysis similar to the one used in several studies [14][17][18][53]. We performed the pRF analysis on two distinct ROIs: BA 6 and IPS. The ROIs were based on the Freesurfer software’s Broadmann and Destrieux atlases. (http://surfer.nmr.mgh.harvard.edu/). For each subject, the pRF analysis was performed on slice time-corrected, realigned, coregistered, and smoothed images.

The idea behind pRF is that neuronal receptive fields are a form of tuning functions that reflect specific stimulus properties. For each subject, pRFs were modeled as one-dimensional Gaussians with two parameters: μ, the stimulus duration, and *σ*, the spread of the pRF.

The pRF tuning function used was a Gaussian changing linearly over time. The reason we chose a linear pRF model is that the durations presented were spaced linearly.

For the pRF modeling, we used the offset of all S1 durations no matter whether S1 was a standard or a comparison duration. This procedure led to the identification of 17 durations (i.e., 0.2. 0.3, 0.4, 0.6, 0.8, 0.9, 1, 1.2, 1.4, 1.5, 1.6, 1.8, 2, 2.1, 2.4, 2.7, and 3 s). For each time point (i.e., each TR) of our fMRI time series and each vertex of the ROIs, the method estimates the overlap between the Gaussian tuning model of a given μ and the presented durations. The combination of Gaussian tuning model and presented duration was convolved to HRF. A coarse-to-fine optimization approach then determined the optimal pRF parameters for which the goodness-of-fit of the predicted time series to the observed data was maximized. Vertices with a goodness of fit, *R*^2^ > 0.1, were included in all further analyses. The maps shown are the projection on the cortical surface of the estimated optimum μ parameter. Different colors represent vertices (i.e., voxels projected onto the cortical surface) selective to different duration ranges.

For the group-level analysis, the pRF maps for each participant were morphed into a common DARTEL template using the morph labels feature of the MNE software (https://mne-tools.github.io/dev/index.html). MNE performs the morphing between subjects using the spherical surfaces provided by Freesurfer. On the DARTEL surfaces, each vertex was assigned the mode of the set of morphed individual data values corresponding to that vertex. This method ensured that each vertex was represented by the most common duration across the subjects.

#### Visualization

For visualization of the group and of the single subject fMRI results in both experiments, we inflated either the DARTEL template (group-level results) or the single-subject T1-weighted image (individual results) using the FreeSurfer pipeline (http://surfer.nmr.mgh.harvard.edu/). To reconstruct surfaces for the DARTEL template, the gray matter and white matter images of the template were combined into a single image with two distinct values assigned to the gray matter and white matter voxels. The combined images were treated as a skullstripped T1-weighted image and submitted to the Freesurfer pipeline for surface reconstruction.

### Quantification of the spatial distribution of chronomaps

#### Surface-based approach

In order to better appreciate the spatial distribution of the chronomaps at the individual level, we identified chronomaps in each single subject by using either the single-subject SPM t-maps (Exp 1) or the pRF maps (Exp 2). The surface-based analyses were performed on images in the subject’s native space.

For a better visualization, these volumetric maps were projected onto the cortical surface of each individual brain. Individual cortical surfaces were reconstructed following the Freesurfer pipeline via segmentation of different brain tissues (projection fraction was set to 0.5).

Individual chronomaps were identified in left SMA and left IPS for Exp 1 and left and right SMA and left and right IPS for Exp 2. In Exp 1, we used anatomical landmarks (i.e., identification of the pre-, postcentral gyri and IPS) to make sure that chronomaps at single-subject level matched the location of those observed at group level.

For Exp 2, the identification of the chronomap at single-subject level was easier since the pRF analysis was performed on two distinct ROIs: BA 6 and IPS. For the identification of SMA chronomap, we took only the medial part of the BA 6.

For each map, we created a surface ROI (left SMA and left IPS for Exp 1 and left, right SMA and left, right IPS for Exp 2), and we manually drew its borders. According to the spatial progression of the maps (from short to long duration-selective voxels) observed at group level, we identified an anterior and a posterior border for SMA maps and a medial and a lateral border for IPS. Those borders were then used as cuts to flatten the surfaces and became the outer edges of the flattened surfaces. For SMA, we took the postcentral gyrus as anatomical landmark for drawing the posterior border.

For each duration-selective vertex and each ROI, we calculated the wRD from one of the borders of the map (D_1_). This border, arbitrarily chosen, was the posterior for SMA map and the lateral for the IPS map.

In more detail, the wRD from the D_1_ border was computed as the following: $wRD=\frac{\sum_{i=1}^{Nvd}w*RD}{Nvd}$, in which *w* is the weight of each vertex defined as the ratio between clustered duration-selective vertices (*Nbrs*) and the total number of vertices maximally responsive to a given duration (*Nvd*), i.e., *w* = *Nnbrs*/*Nvd*. Whereas *RD* was the ratio between the distance from one of the borders (*D*_1_) and the mean distance between the two borders (*TD*)*RD* = *D*_1_/*TD*.

For each map, we computed the wRD of each duration-selective cluster, and we identified a slope of their spatial progression. The individual slopes were used to perform a Wilcoxon test in order to check the statistical significance of the spatial progression of the maps.

#### Volume-based approach

To make sure that the results from the surface-based analyses depicted reality and were not the product of wrong projection of voxels onto the surface, we also performed volume-based analyses.

Similar to surface-based analysis, here, we also identified for each experiment and each subject chronomaps in SMA and IPS.

Also, for volumetric maps, we defined maps’ borders. These were anterior and posterior for SMA and medial and lateral for IPS.

In order to check whether the duration-selective voxels followed the same spatial progression as the surface maps, we identified for each subject and each map the “preferred duration” of different portions of the map. More precisely, we binned the individual volumetric ROIs (in the subject’s native space) in parallel planes of 1.5-mm width. Within each volumetric bin, the “preferred duration” was calculated as the duration the majority of activated voxels responded to. Thus, for each subject, we had a sequence of preferred durations between the two borders of the map. We then decided to compute the average of preferred durations across subjects. Since different subjects had sequences of preferred durations of different length, we decided to proceed as follows: we calculated for each spatial bin its relative distance from one border (D1) of the map (i.e., posterior for SMA and lateral for IPS). Then for each map, we created a single sequence of “preferred durations,” which included the sequences of all subjects ordered according to their relative distance from D1. In order to reduce the total length of this long sequence, we averaged every five values of the sequence. The result of this procedure is displayed in panel C of Fig 3 and panel A of Figs 7 and 8.

In order to appreciate the spatial distribution of the maps at single-subject level, for each subject and each duration-selective cluster of voxels, we also estimated the wCntrs. wCntrs were estimated in individual maps normalized to a common Dartel space (Dartel-11 and Dartel-10 for Exp.1 and Exp.2 respectively). Within a cluster of duration-selective voxels, every voxel was assigned a weight based on the number of neighboring voxels with the same duration selectivity. This means that clustered voxels had more weight than sparse ones. The wCntrs were then calculated by taking into account the position of all duration-selective voxels within a cluster, but each position was represented as many times as the weight assigned to a specific voxel. This measure allowed us to visualize in a single graph the central position of all duration-selective clusters in all subjects (see panel B of Figs 3, 7 and 8).

### Tuning analysis

To check the response properties of duration-selective voxels, we looked at the BOLD response to preferred and nonpreferred durations. In both experiments, for each subject and each cluster of duration-selective voxels within the different chronomaps (i.e., SMA and IPS in the left hemisphere for Exp 1 and left and right SMA for Exp 2), we looked at the normalized hemodynamic response to preferred and nonpreferred durations.

$$\mathrm{The}\;\mathrm{normalization}\;\mathrm{was}\;\mathrm{performed}\;\mathrm{as}\;\mathrm{follows}:\;BOLD(t)=\frac{\sum_{i=1}^{Nruns}\sum_{v=1}^{Nvoxels(}\frac{x(t)-MB)/MB}{Nvoxels}}{Nruns*std(\sum_{v=1}^{Nvoxels(}\frac{x(t)-MB)/MB}{Nvoxels})}$$

in which t is the signal in a given voxel and MB is the baseline obtained averaging the signal of t across runs. Normalization was then performed by subtracting the signal in a given voxel from a baseline value and dividing it by the baseline. The BOLD response was aligned to the second volume (i.e., a TR) after the offset of the S1 duration. Within a single subject, we first averaged the BOLD signal across the voxels of a cluster and then across the fMRI runs.

### Check for venous artifacts

In order to rule out the contribution of venous voxels to SMA chronomaps, we used two distinct methods for the two experiments. In Exp 1, we reasoned that any “venous” voxels in SMA that contribute to the map should have very low signal intensity and a very high z score. As you can see from S18 Fig (S4 Data), in which we show for each subject and each duration-selective voxel the z scores (x-axis) plotted against the mean signal intensity across the 18 runs, none of the voxels had a very high z score and a very low signal intensity.

In Exp 2, we reasoned that a “venous vertex” should not show any duration tuning, i.e., it should be a vertex with a tuning function characterized by an abnormally wide spread (σ). In S19 and S20 Figs (see S3 Data), we show for each subject, for the left and right SMA, in the temporal and in the orientation task, the correlations between σ and μ. Again, none of the vertices had a very wide spread; actually, all σ were <1. The reason to perform such correlation was also to check the existence of a systematic relationship in the tuning function of mean and spread for the different duration-selective vertices (e.g., small σ for small μ). Unfortunately, we failed to find the existence of any relationship.

## Supporting information

S1 Table Stereotaxic Dartel-11 coordinates (mm) for regions activated at the offset of the four S1 durations. Voxels activated at *P* < 0.05, FWE cluster-level corrected for multiple comparisons across the entire brain volume. FWE, familywise error.
(XLSX)

S1 Fig fMRI group results from Exp 1. Activations correlated with the offset of the four different S1 durations (*P* < 0.05, FWE cluster-level corrected for multiple comparisons across the whole brain). The significant clusters are overlaid on a high-resolution MP2RAGE normalized to the Dartel-11 template. fMRI, functional magnetic resonance imaging; FWE, familywise error; MP2RAGE, magnetization prepared rapid gradient echo.
(TIF)

S2 Fig fMRI results, individual data (*N* = 11) from Exp 1, left SMA (L). For each subject, we show the brain map and the wRD from the P border. Individual maps were obtained using a winner-take-all procedure based on statistical t-maps. We computed four different t-maps for each of the four S1 durations (*P*_FWE_-cluster level < 0.05, corrected for multiple comparisons across the whole brain). The clusters of voxels maximally responsive to each of the S1 durations were then projected onto individual subjects’ flattened surfaces. The individual A and P borders are shown with white vertical lines. In the plot, the colored diamonds represent the duration-selective vertices (x-axis) plotted according to their wRD from the P border of the map. The white line in each plot is the result of a fitting procedure that helps to identify the spatial progression of the maps. The durations of the color bar are red = 0.2, orange = 0.4, yellow = 0.6, and green = 1s. The data can be found in S4 Data. A, anterior; fMRI, functional magnetic resonance imaging; FWE, familywise error; P, posterior; PCG, precentral gyrus; S1, first stimulus; SMA, supplementary motor area; wRD, weighted relative distance.
(TIF)

S3 Fig fMRI results, wRD slopes from Exp 1 Individual slopes obtained by fitting the wRD from the posterior border of the four duration-selective clusters. For left SMA and left IPS, we plotted the individual slopes (black lines) and the average slopes (red line). *P < 0.01. The data can be found in S3 Data. fMRI, functional magnetic resonance imaging; IPS, intraparietal sulcus; SMA, supplementary motor area; wRD, weighted relative distance.
(TIF)

S4 Fig fMRI results, individual data (*N* = 11) of Exp 1 for the left IPS. For each subject, we show the brain map in the temporal task and the wRD from the L border. Individual maps were obtained using a winner-take-all procedure based on statistical t-maps. We computed four different t-maps for each of the four S1 durations (*P*_FWE_-cluster level < 0.05, corrected for multiple comparisons across the whole brain). The clusters of voxels maximally responsive to each of the S1 durations were then projected onto flattened surfaces in the subjects’ native space. The individual M and L borders are shown with white vertical lines. In the plot, the colored diamonds represent the duration-selective vertices (x-axis) plotted according to their wRD from the L border of the map. The white line in each plot is the result of a fitting procedure that helps to identify the spatial progression of the maps. The durations of the color bar are red = 0.2, orange = 0.4, yellow = 0.6, and green = 1 s. The data can be found in S4 Data. fMRI, functional magnetic resonance imaging; FWE, familywise error; IPS, intraparietal sulcus; L, lateral; M, medial; S1, first stimulus; wRD, weighted relative distance.
(TIF)

S5 Fig fMRI results, spatial progression of duration-selective vertices in IPS. (A) Here, we show the group median (biggest colored diamonds) and the full distribution of individual data (smaller diamonds) of the wRDs of duration-selective vertices from the L border of the chronomap. wRDs were first computed for each individual subject on chronomaps overlaid on flattened surfaces in participants’ native space. (B) 2D projection of wCntrs in the y-z plane for the duration-selective voxels. Different colors indicate voxels with different duration selectivity; diamonds with the same color represent the different subjects (*n* = 11). This value differs across duration conditions because not all subjects had the full range of duration-selective voxels. The data can be found in S3 Data. fMRI, functional magnetic resonance imaging; IPS, intraparietal sulcus; L, lateral; wCntr, weighted centroid; wRD, weighted relative distance.
(TIF)

S6 Fig BOLD time course from Exp 1. Normalized signal change of the shortest- (red line) and longest- (green line) duration–selective clusters over the trial period, when either a shortest or a longest duration was presented. On the x-axis, 1 = S1 offset, 8 = onset of the following trial. TR = 1.3 sec. As expected, after stimulus offset, the hemodynamic response rose at a similar time in the two clusters for the two durations (approximately second TR after stimulus offset); the signal had a greater amplitude for the appropriate pair of stimulus and duration-selective clusters, e.g., the 0.2 s duration-selective cluster when the 0.2 s stimulus was presented. The data can be found in S3 Data. BOLD, blood oxygenation level-dependent; S1, first stimulus; TR, repetition time.
(TIF)

S7 Fig Duration tuning in IPS from Exp 1. (A) Group average of normalized BOLD responses (y-axis) of duration-selective voxels (different lines are different duration-selective voxels) for preferred and nonpreferred durations. The four presented durations are in the x-axis. The BOLD signal in the duration-selective voxels is aligned to the presentation timings of the different duration ranges (i.e., second volume after S1 offset). The colored diamonds represent the point in time when the hemodynamic response of duration-selective voxels matched the presentation timing of the appropriate duration (e.g., red-labeled voxels when the shortest S1 duration is presented). The color code is as in Fig 2. Normalization was performed first in each individual subject to the mean signal intensity across fMRI runs and then for each duration-selective cluster to the signal associated to the preferred duration. (B) Normalized BOLD response to PD, neighboring (PD ± 1), and distant durations (PD ± 2) averaged across subjects and duration-selective voxels. The data can be found in S3 Data. Error bars are standard errors. BOLD, blood oxygenation level-dependent; fMRI, functional magnetic resonance imaging; IPS, intraparietal sulcus; PD, preferred duration; S1, first stimulus.
(TIF)

S8 Fig fMRI results, individual data (*N* = 10) of Exp 2 for the left (L) SMA in the time task. For each subject, we show the brain map and the wRD from the posterior border. Individual maps were obtained using the pRF method. We used as pRF models a one-dimensional Gaussian curve with two parameters: μ, the stimulus duration and σ, the spread of the pRF. Here we show the estimated μ on the cortical surface (medial part of BA6) of the estimated μ parameter. Different colors represent vertices (i.e., voxels projected onto the cortical surface) selective to different duration ranges (i.e., vertices with different estimated μ*)*. The individual anterior (A) and posterior (P) borders are shown with white vertical lines. In the plot the colored diamonds represent the duration-selective vertices (x-axis) plotted according to their wRD from the posterior border of the map. The white line in each plot is the result of a fitting procedure that helps to identify the spatial progression of the maps. The data can be found in S4 Data. The slope is calculated separately for durations below and above 1 second. fMRI, functional magnetic resonance imaging; PCG, precentral gyrus; SMA, supplementary motor area wRD, weighted relative distance.
(TIF)

S9 Fig fMRI results, individual data (N = 10) of Exp 2 for the right (R) SMA in the time task. For the description of the figure see legend S8 Fig. The data can be found in S4 Data. fMRI, functional magnetic resonance imaging; SMA, supplementary motor area.
(TIF)

S10 Fig fMRI results, individual data (*N* = 10) of Exp 2 for the left (L) SMA in the orientation task. For the description of the figure see legend S8 Fig. The data can be found in S4 Data. fMRI, functional magnetic resonance imaging; L, left; SMA, supplementary motor area.
(TIF)

S11 Fig fMRI results, individual data (*N* = 10) of Exp 2 for the right (R) SMA in the orientation task. For the description of the figure, see legend S8 Fig. The data can be found in S4 Data. fMRI, functional magnetic resonance imaging; SMA, supplementary motor area.
(TIF)

S12 Fig fMRI results, wRD slopes from Exp 2. Individual slopes obtained by fitting the wRD from the posterior border of the 17 duration-selective clusters. For left and right SMA in the time and in the orientation task, we plotted the individual slopes (black lines) and the average slopes (red line). **P < 0.001, *P < 0.01. The data can be found in S3 Data. fMRI, functional magnetic resonance imaging; SMA, supplementary motor area; wRD, weighted relative distance.
(TIF)

S13 Fig pRF group-level results of Exp 2. Here we show the estimated μ on the cortical surface for the IPS of both hemisphere of the estimated μ parameter. Different colors represent vertices (i.e., voxels projected onto the cortical surface) selective to different duration ranges (i.e., vertices with different estimated μ). We show the results of the group (average of 10 subjects) for the 17 estimated μ. The 17 μ are the 17 durations presented in the 10 different trial type (either S1 or S2). The color scale goes from red, i.e., shortest duration (0.2 s) to dark blue, i.e., longest duration (3 s). The white lines give an example of the map borders as they were drawn to estimate the wRD in the individual subjects. On the left-hand side, time maps in time task, on the right-hand side time maps in the orientation task. The data can be found in S4 Data. L, left; R, right; CS, central sulcus; IPS, intraparietal sulcus; L, lateral; M, medial; wRD, weighted relative distance.
(TIF)

S14 Fig BOLD time course from Exp 2. Normalized signal change of the shortest- (red line) and the longest- (blue line) duration–selective clusters of voxels over the time of a cycle (i.e., 44 seconds = 22 TRs) in left and right SMA for time and orientation tasks. For each subject, we averaged the signal across 20 cycles. Please note that the signal in descending cycles was swapped to match the ascending ones. The data can be found in S3 Data. BOLD, blood oxygenation level-dependent; SMA, supplementary motor area; TR repetition time.
(TIF)

S15 Fig Correlations between spatial progression of the maps and temporal performance in Exp 1. The two scatterplots show the correlations (using Kendal's tau correlation coefficient) between the individual slopes of the wRD in left SMA with two behavioral indexes of temporal performance: accuracy (left panel) and coefficient of variation (i.e., CV = standard deviation/duration, right panel). The data can be found in S3 Data. SMA, supplementary motor area; wRD, weighted relative distance.
(TIF)

S16 Fig Correlations between spatial progression of the maps and temporal performance in Exp 2. The scatterplots show the correlations (using Kendal's tau correlation coefficient) between the individual slopes of the wRD measured for left and right SMA, with two behavioral indexes of temporal performance: accuracy (upper panel) and coefficient of variation (i.e., CV = standard deviation/duration, lower panel). The data can be found in S3 Data. SMA, supplementary motor area; wRD, weighted relative distance.
(TIF)

S17 Fig Size of the different duration-selective clusters in Exp 1 and Exp 2. For both experiments, we show the mean and standard error of the proportions (i.e., number of vertices of a given type/total number of vertices in the map) of different duration-selective vertices within the SMA chronomaps. For Exp 1, we show SMA left (A). For Exp 2, we show the average of left and right SMA maps for the time and the orientation tasks (B). The data can be found in S3 Data. SMA, supplementary motor area
(TIF)

S18 Fig Check for venous artifact in Exp 1. The scatterplots show for each subject and each duration-selective voxel the z scores (x-axis) plotted against the mean signal intensity across the 18 runs. None of the voxels had a very high z score and a very low signal intensity. The data can be found in S4 Data.
(TIF)

S19 Fig Check for venous artifact in the temporal task of Exp 2. The plots show the correlations between σ and μ for the left (blue) and the right (red) SMA. Each plot is a subject. None of the voxels has a very wide spread; actually, all σ are <1. The data can be found in S3 Data. SMA, supplementary motor area.
(TIF)

S20 Fig Check for venous artifact in the orientation task of Exp 2. The plots show the correlations between σ and μ for the left (blue) and the right (red) SMA. Each plot is a subject. None of the voxels has a very wide spread (all μ are <1). The data can be found in S3 Data. SMA, supplementary motor area.
(TIF)

S1 Data Excel spreadsheet containing, in separate sheets, the underlying numerical data and statistical analysis for Figs 1B, 3A, 3B, 3C, 4A, 4B, 5B, 7A, 7B, 7C, 8A, 8B, 8C, 9 and 11B. (XLSX)

S2 Data Matlab structure containing the underlying numerical data for Figs 2, 6 and 10. (MAT)

S3 Data Excel spreadsheet containing, in separate sheets, the underlying numerical data and statistical analysis for S3, S5A, S5B, S6, S7A, S7B, S12, S14, S15, S16, S17A, S17B, S19 and S20 Figs. (XLSX)

S4 Data Matlab structure containing the underlying numerical data for S2, S4, S8, S9, S10, S11, S13 and S18 Figs. (MAT)

## Abbreviations

7T: 7-Tesla
BOLD: blood oxygenation level-dependent
fMRI: functional magnetic resonance imaging
FWE: familywise error
GLM: General Linear Model
HRF: hemodynamic response function
IPL: inferior parietal lobule
IPS: intraparietal sulcus
PD: preferred duration
pRF: population Receptive Field
ROI: region of interest
S1: first stimulus
S2: second stimulus
SMA: supplementary motor area
SOA: stimulus onset asynchrony
TMS: Transcranial Magnetic Stimulation
TR: repetition time
wCntr: weighted centroid; wRD, weighted relative distance
