# Supplementary figures and images for: Chronotopic maps in human supplementary motor area
Source: PLoS Biol. 2019 Mar 21;17(3):e3000026. doi: 10.1371/journal.pbio.3000026 (PMC6428248; doi:10.1371/journal.pbio.3000026)

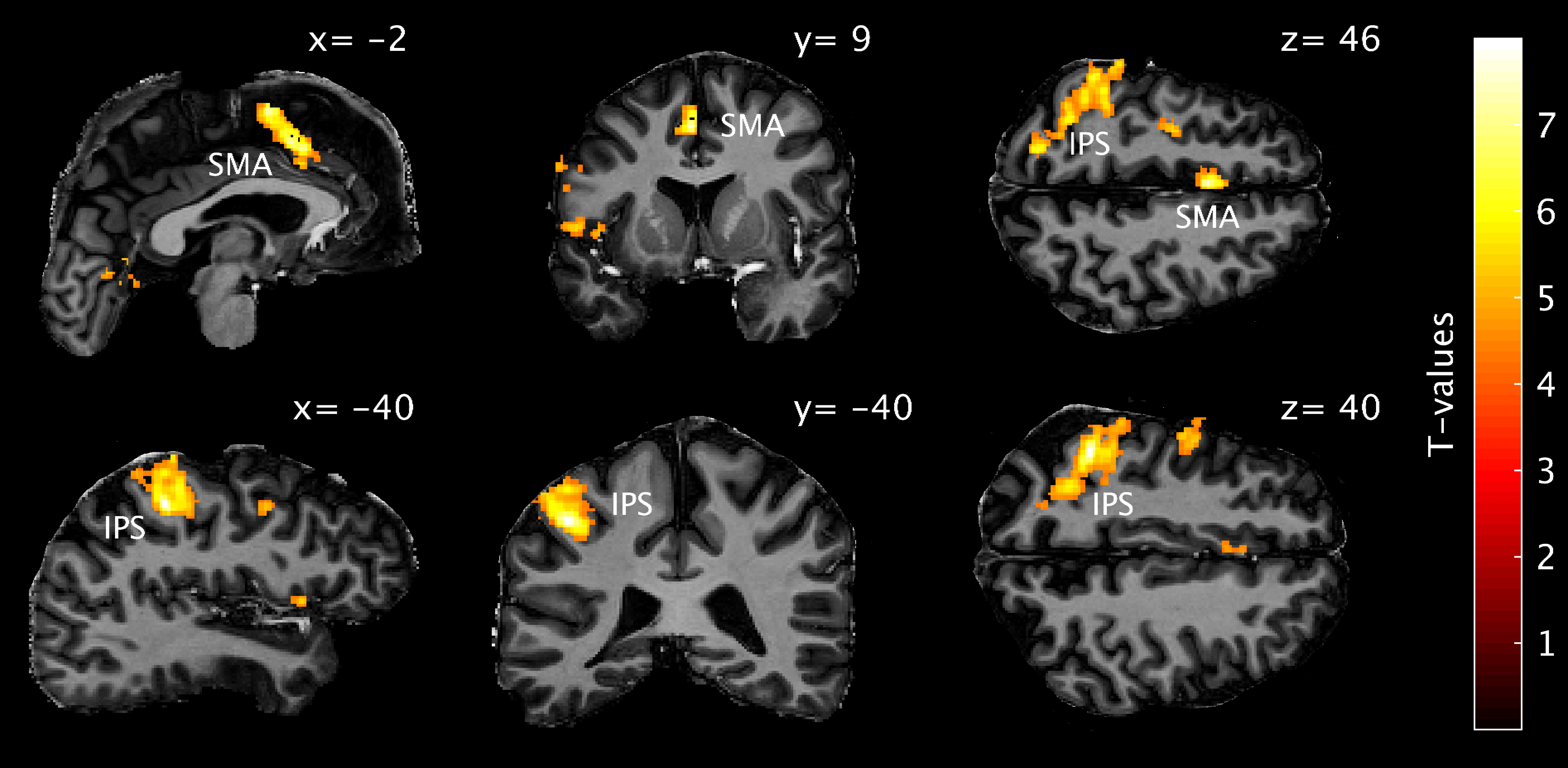

Supplement: S1 Fig — Activations correlated with the offset of the four different S1 durations (P < 0.05, FWE cluster-level corrected for multiple comparisons across the whole brain). The significant clusters are overlaid on a high-resolution MP2RAGE normalized to the Dartel-11 template. fMRI, functional magnetic resonance imaging; FWE, familywise error; MP2RAGE, magnetization prepared rapid gradient echo. (TIF) [file pbio.3000026.s002.tif]

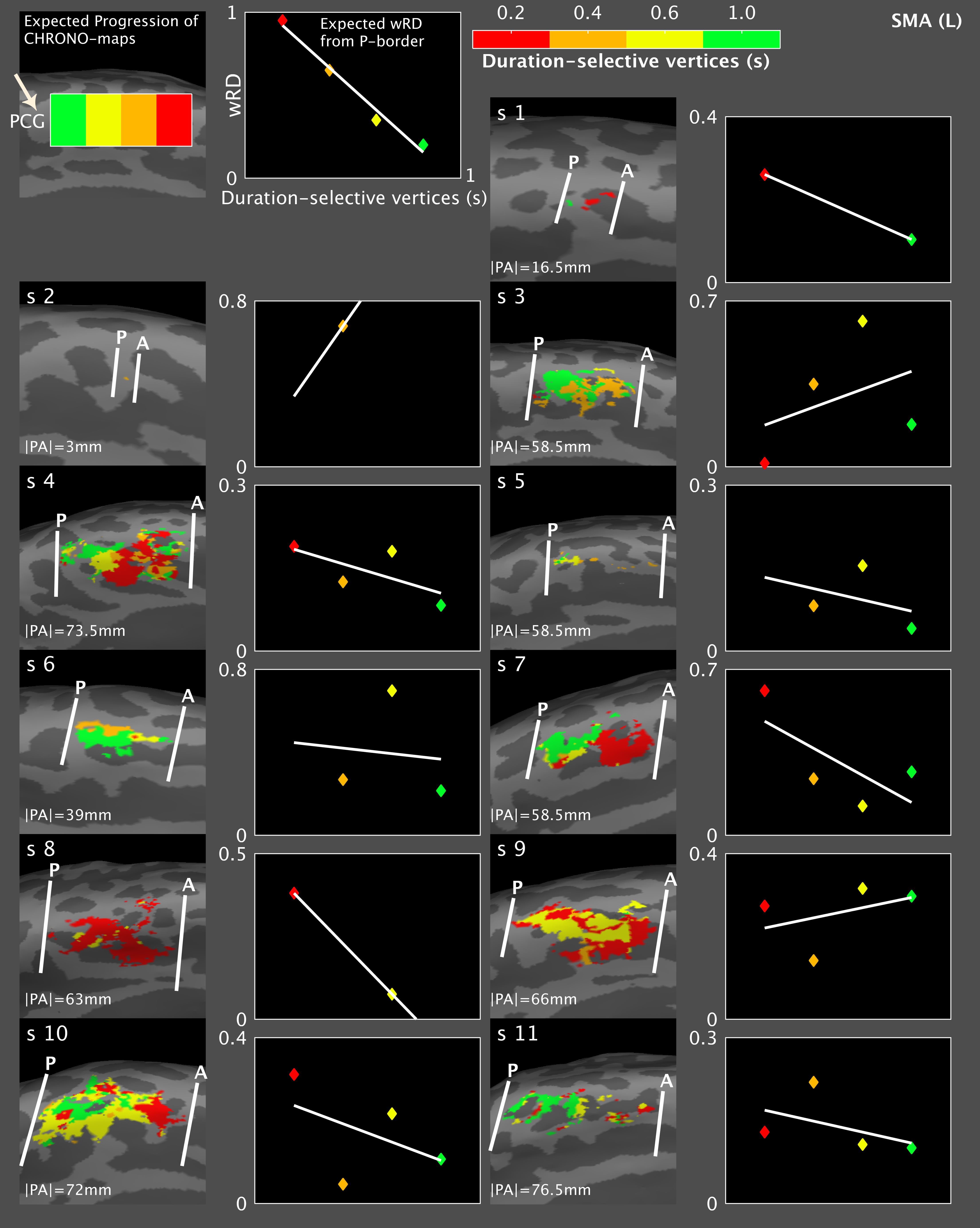

Supplement: S2 Fig — For each subject, we show the brain map and the wRD from the P border. Individual maps were obtained using a winner-take-all procedure based on statistical t-maps. We computed four different t-maps for each of the four S1 durations (PFWE-cluster level < 0.05, corrected for multiple comparisons across the whole brain). The clusters of voxels maximally responsive to each of the S1 durations were then projected onto individual subjects’ flattened surfaces. The individual A and P borders are shown with white vertical lines. In the plot, the colored diamonds represent the duration-selective vertices (x-axis) plotted according to their wRD from the P border of the map. The white line in each plot is the result of a fitting procedure that helps to identify the spatial progression of the maps. The durations of the color bar are red = 0.2, orange = 0.4, yellow = 0.6, and green = 1s. The data can be found in S4 Data. A, anterior; fMRI, functional magnetic resonance imaging; FWE, familywise error; P, posterior; PCG, precentral gyrus; S1, first stimulus; SMA, supplementary motor area; wRD, weighted relative distance. (TIF) [file pbio.3000026.s003.tif]

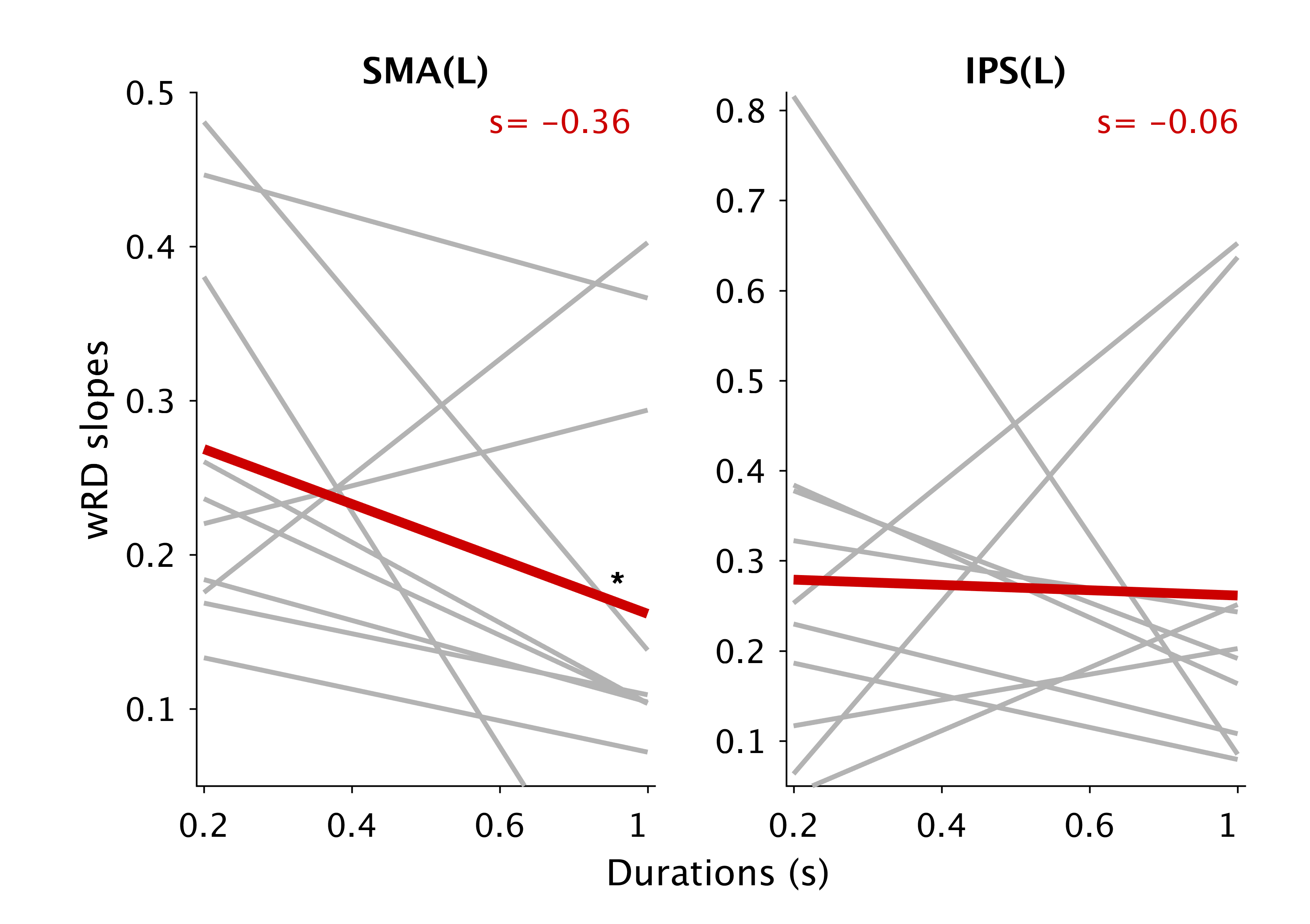

Supplement: S3 Fig — For left SMA and left IPS, we plotted the individual slopes (black lines) and the average slopes (red line). *P < 0.01. The data can be found in S3 Data. fMRI, functional magnetic resonance imaging; IPS, intraparietal sulcus; SMA, supplementary motor area; wRD, weighted relative distance. (TIF) [file pbio.3000026.s004.tif]

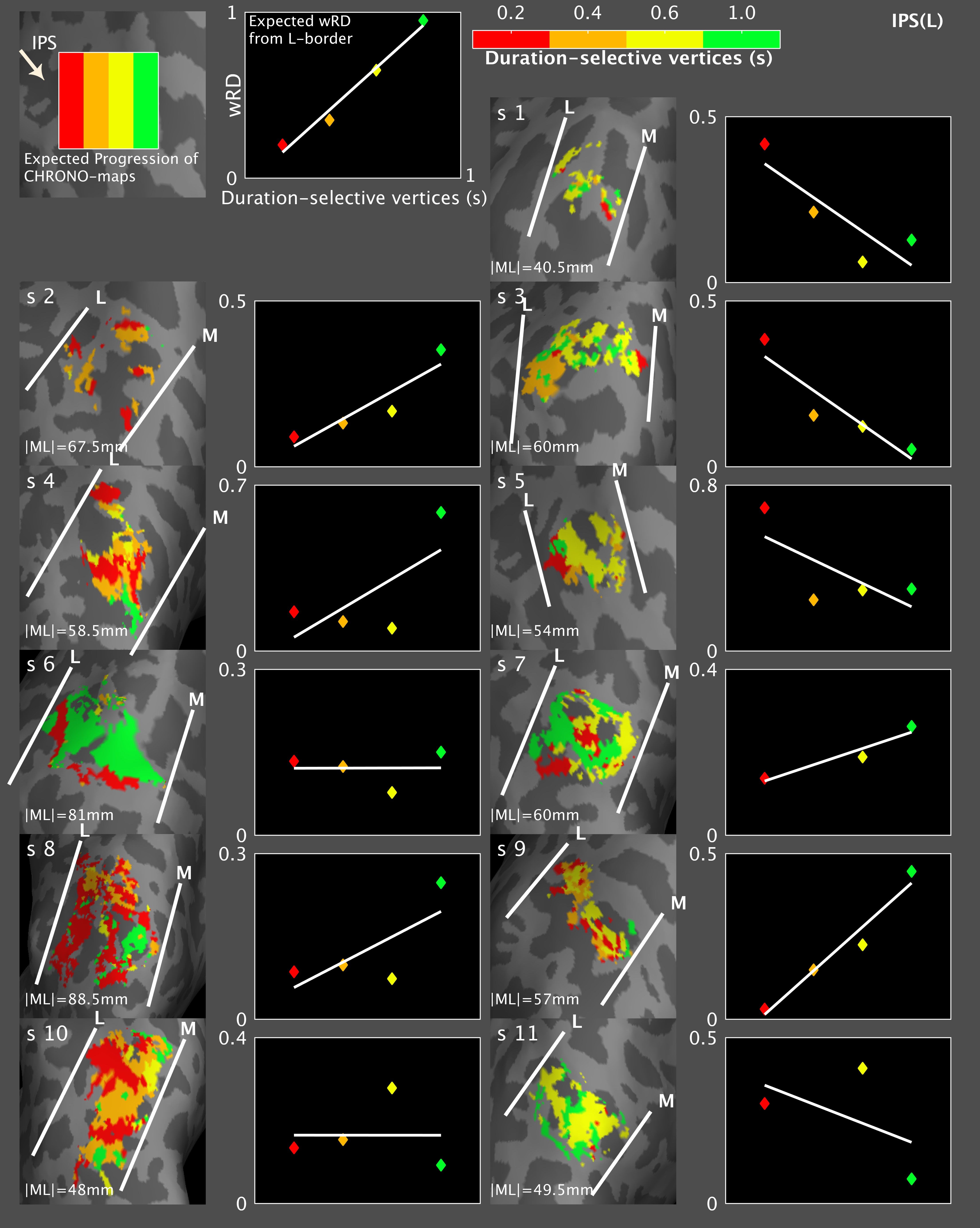

Supplement: S4 Fig — For each subject, we show the brain map in the temporal task and the wRD from the L border. Individual maps were obtained using a winner-take-all procedure based on statistical t-maps. We computed four different t-maps for each of the four S1 durations (PFWE-cluster level < 0.05, corrected for multiple comparisons across the whole brain). The clusters of voxels maximally responsive to each of the S1 durations were then projected onto flattened surfaces in the subjects’ native space. The individual M and L borders are shown with white vertical lines. In the plot, the colored diamonds represent the duration-selective vertices (x-axis) plotted according to their wRD from the L border of the map. The white line in each plot is the result of a fitting procedure that helps to identify the spatial progression of the maps. The durations of the color bar are red = 0.2, orange = 0.4, yellow = 0.6, and green = 1 s. The data can be found in S4 Data. fMRI, functional magnetic resonance imaging; FWE, familywise error; IPS, intraparietal sulcus; L, lateral; M, medial; S1, first stimulus; wRD, weighted relative distance. (TIF) [file pbio.3000026.s005.tif]

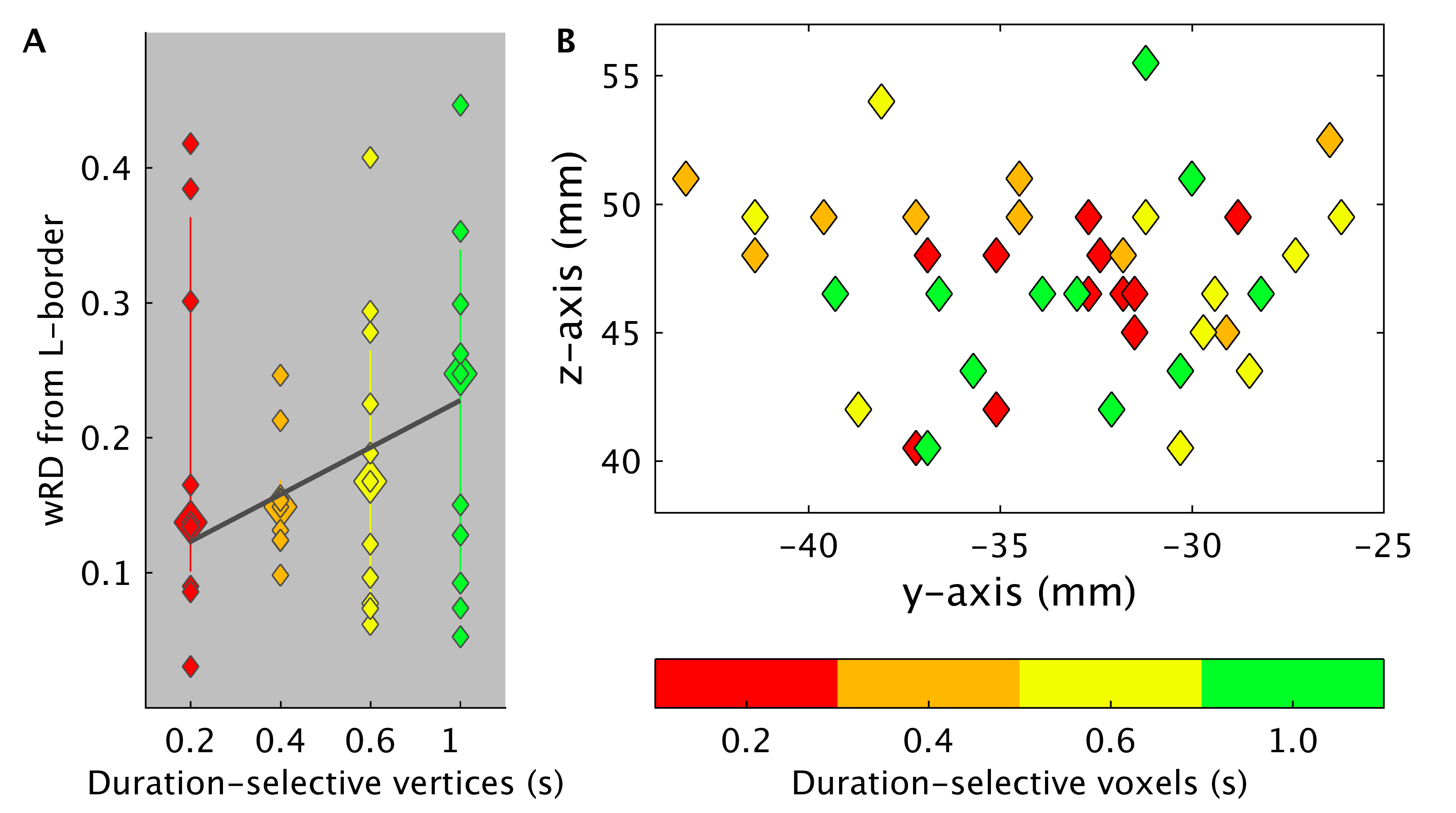

Supplement: S5 Fig — (A) Here, we show the group median (biggest colored diamonds) and the full distribution of individual data (smaller diamonds) of the wRDs of duration-selective vertices from the L border of the chronomap. wRDs were first computed for each individual subject on chronomaps overlaid on flattened surfaces in participants’ native space. (B) 2D projection of wCntrs in the y-z plane for the duration-selective voxels. Different colors indicate voxels with different duration selectivity; diamonds with the same color represent the different subjects (n = 11). This value differs across duration conditions because not all subjects had the full range of duration-selective voxels. The data can be found in S3 Data. fMRI, functional magnetic resonance imaging; IPS, intraparietal sulcus; L, lateral; wCntr, weighted centroid; wRD, weighted relative distance. (TIF) [file pbio.3000026.s006.tif]

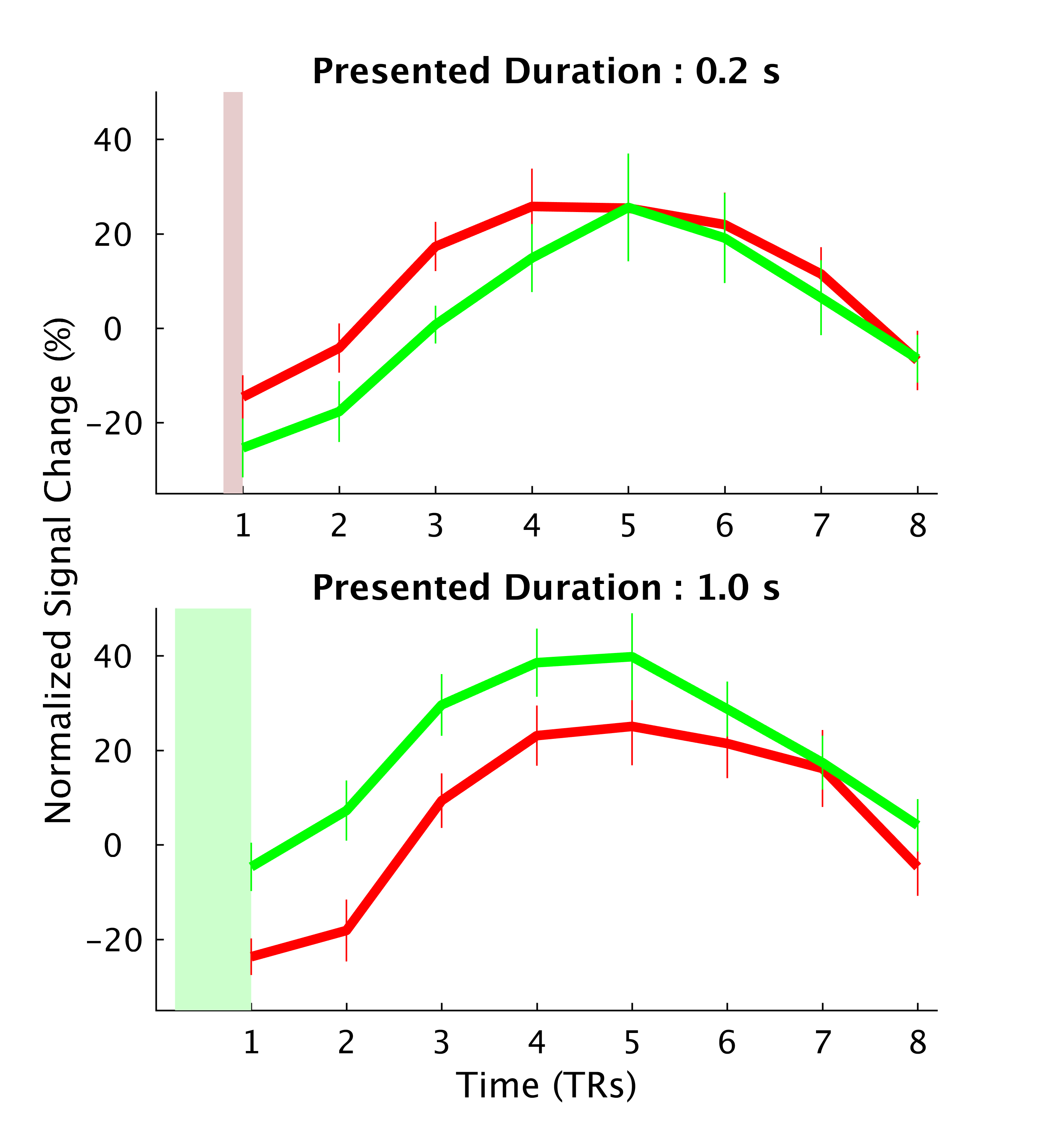

Supplement: S6 Fig — Normalized signal change of the shortest- (red line) and longest- (green line) duration–selective clusters over the trial period, when either a shortest or a longest duration was presented. On the x-axis, 1 = S1 offset, 8 = onset of the following trial. TR = 1.3 sec. As expected, after stimulus offset, the hemodynamic response rose at a similar time in the two clusters for the two durations (approximately second TR after stimulus offset); the signal had a greater amplitude for the appropriate pair of stimulus and duration-selective clusters, e.g., the 0.2 s duration-selective cluster when the 0.2 s stimulus was presented. The data can be found in S3 Data. BOLD, blood oxygenation level-dependent; S1, first stimulus; TR, repetition time. (TIF) [file pbio.3000026.s007.tif]

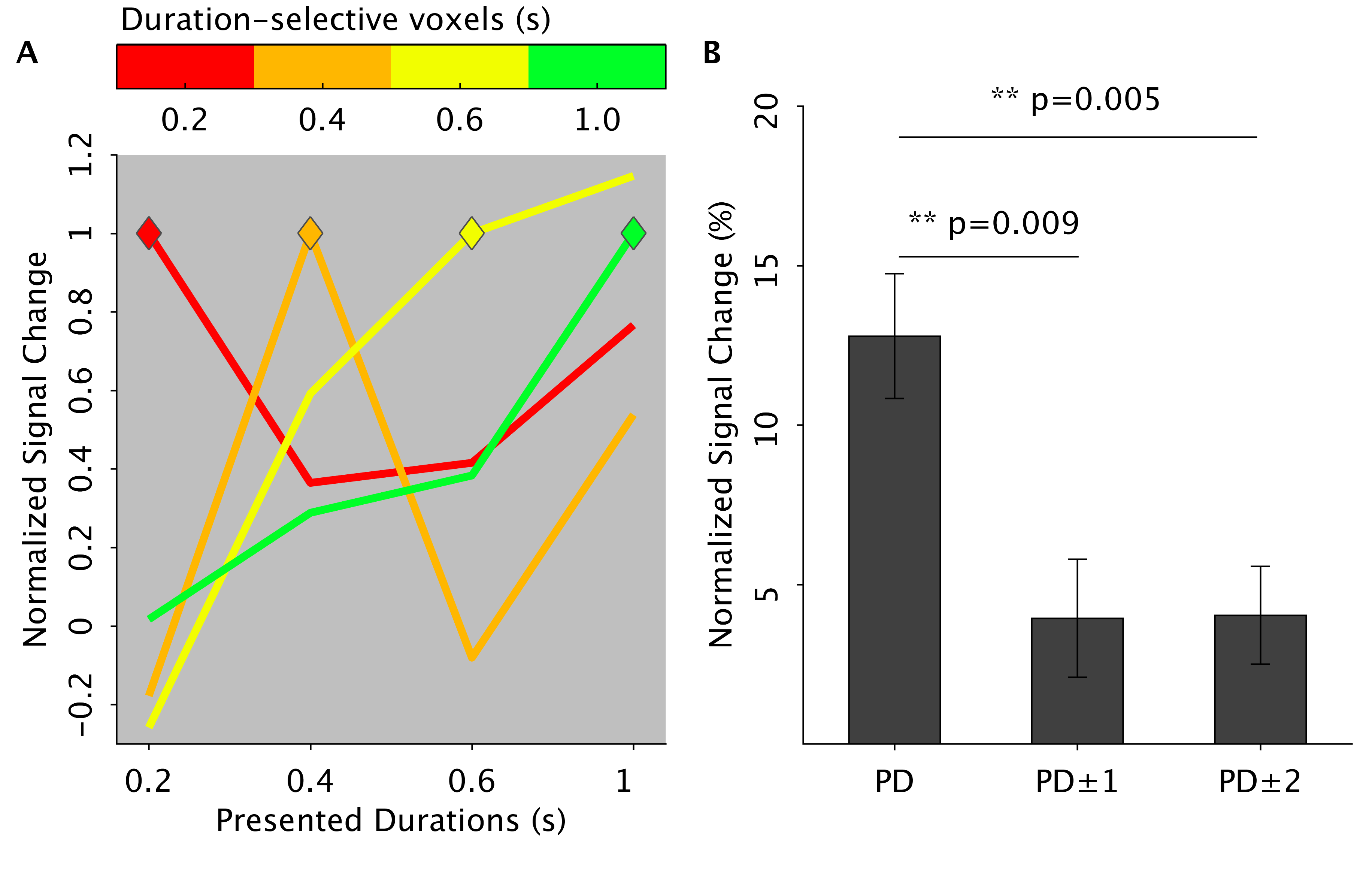

Supplement: S7 Fig — (A) Group average of normalized BOLD responses (y-axis) of duration-selective voxels (different lines are different duration-selective voxels) for preferred and nonpreferred durations. The four presented durations are in the x-axis. The BOLD signal in the duration-selective voxels is aligned to the presentation timings of the different duration ranges (i.e., second volume after S1 offset). The colored diamonds represent the point in time when the hemodynamic response of duration-selective voxels matched the presentation timing of the appropriate duration (e.g., red-labeled voxels when the shortest S1 duration is presented). The color code is as in Fig 2. Normalization was performed first in each individual subject to the mean signal intensity across fMRI runs and then for each duration-selective cluster to the signal associated to the preferred duration. (B) Normalized BOLD response to PD, neighboring (PD ± 1), and distant durations (PD ± 2) averaged across subjects and duration-selective voxels. The data can be found in S3 Data. Error bars are standard errors. BOLD, blood oxygenation level-dependent; fMRI, functional magnetic resonance imaging; IPS, intraparietal sulcus; PD, preferred duration; S1, first stimulus. (TIF) [file pbio.3000026.s008.tif]

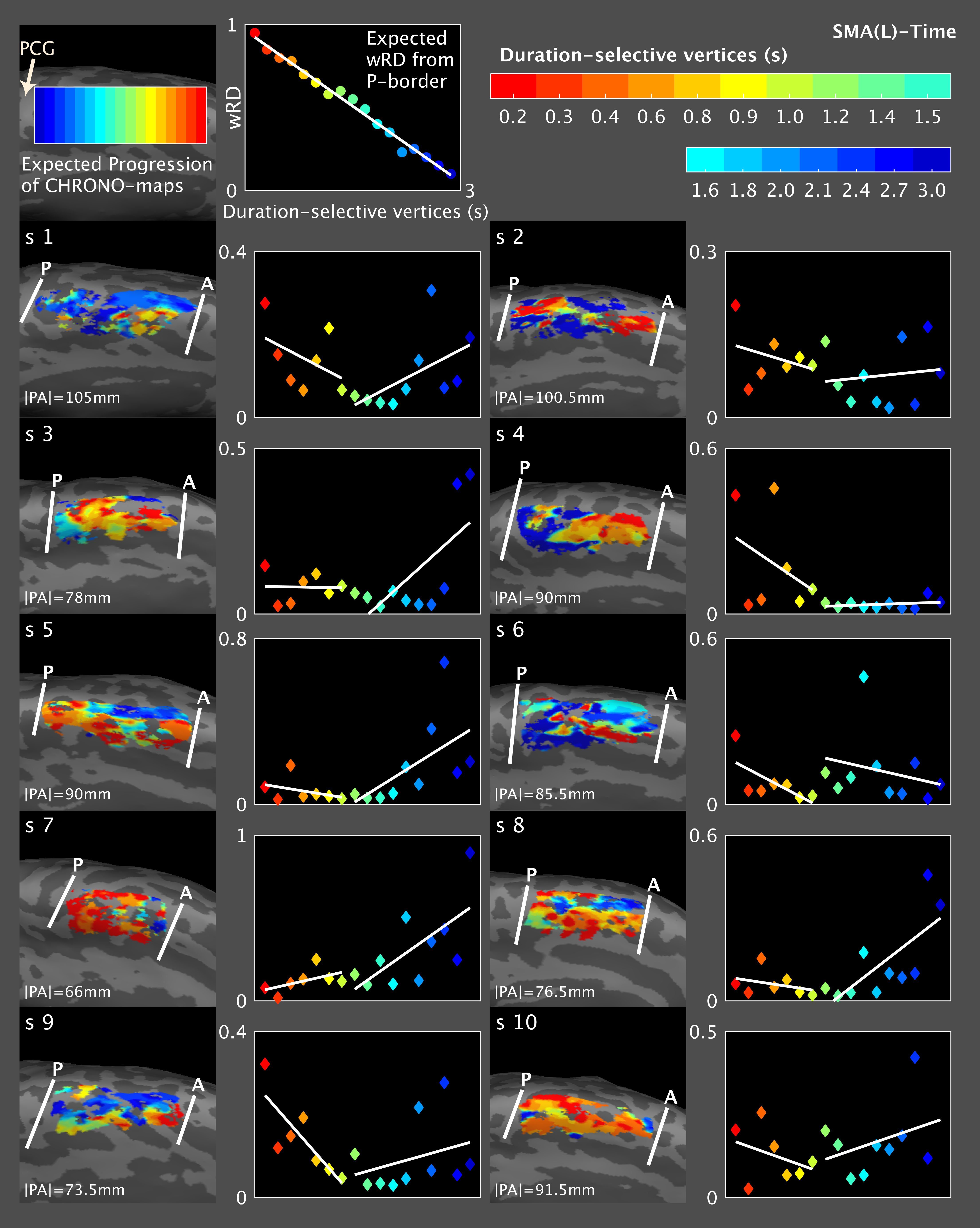

Supplement: S8 Fig — For each subject, we show the brain map and the wRD from the posterior border. Individual maps were obtained using the pRF method. We used as pRF models a one-dimensional Gaussian curve with two parameters: μ, the stimulus duration and σ, the spread of the pRF. Here we show the estimated μ on the cortical surface (medial part of BA6) of the estimated μ parameter. Different colors represent vertices (i.e., voxels projected onto the cortical surface) selective to different duration ranges (i.e., vertices with different estimated μ). The individual anterior (A) and posterior (P) borders are shown with white vertical lines. In the plot the colored diamonds represent the duration-selective vertices (x-axis) plotted according to their wRD from the posterior border of the map. The white line in each plot is the result of a fitting procedure that helps to identify the spatial progression of the maps. The data can be found in S4 Data. The slope is calculated separately for durations below and above 1 second. fMRI, functional magnetic resonance imaging; PCG, precentral gyrus; SMA, supplementary motor area wRD, weighted relative distance. (TIF) [file pbio.3000026.s009.tif]

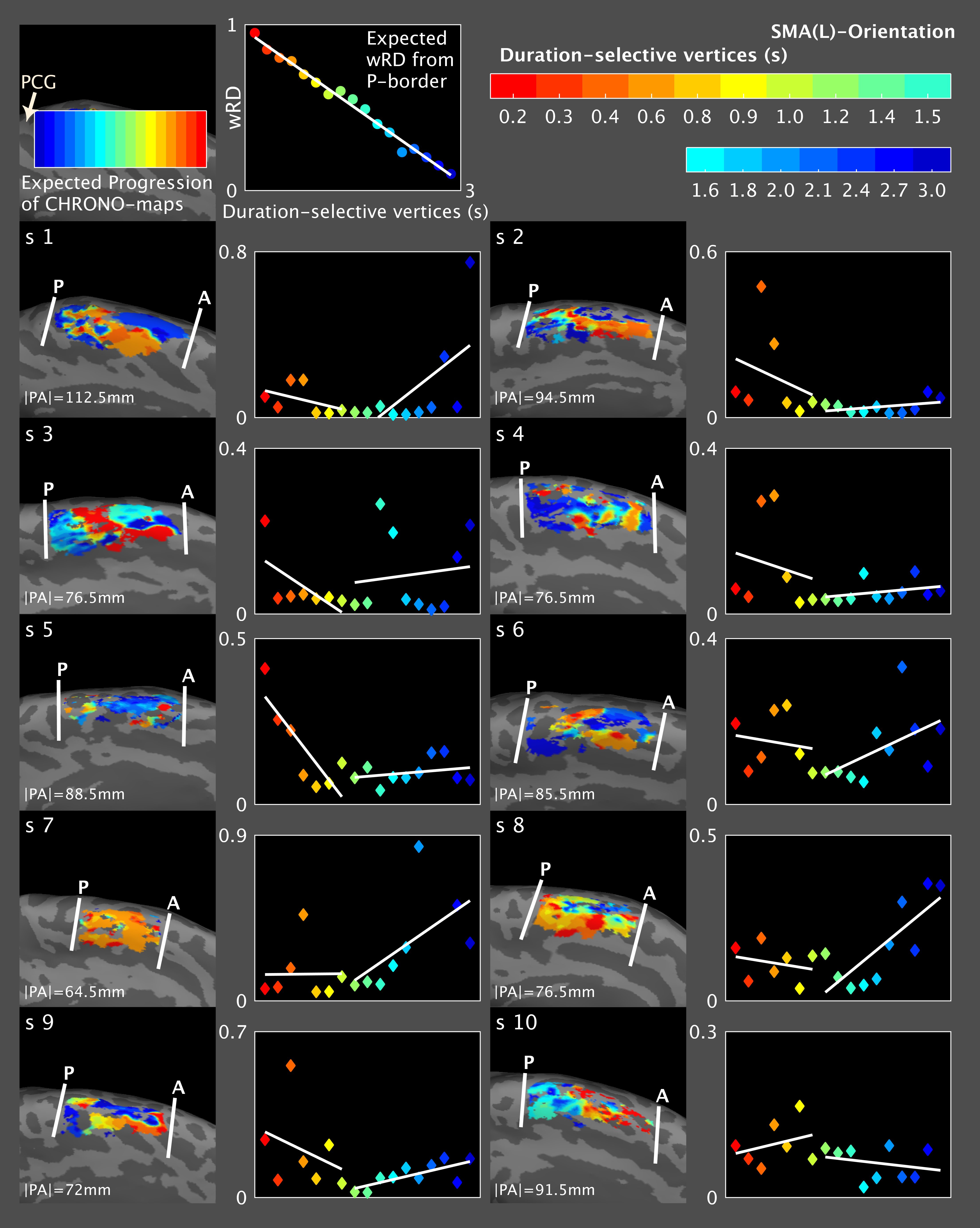

Supplement: S9 Fig — For the description of the figure see legend S8 Fig. The data can be found in S4 Data. fMRI, functional magnetic resonance imaging; SMA, supplementary motor area. (TIF) [file pbio.3000026.s010.tif]

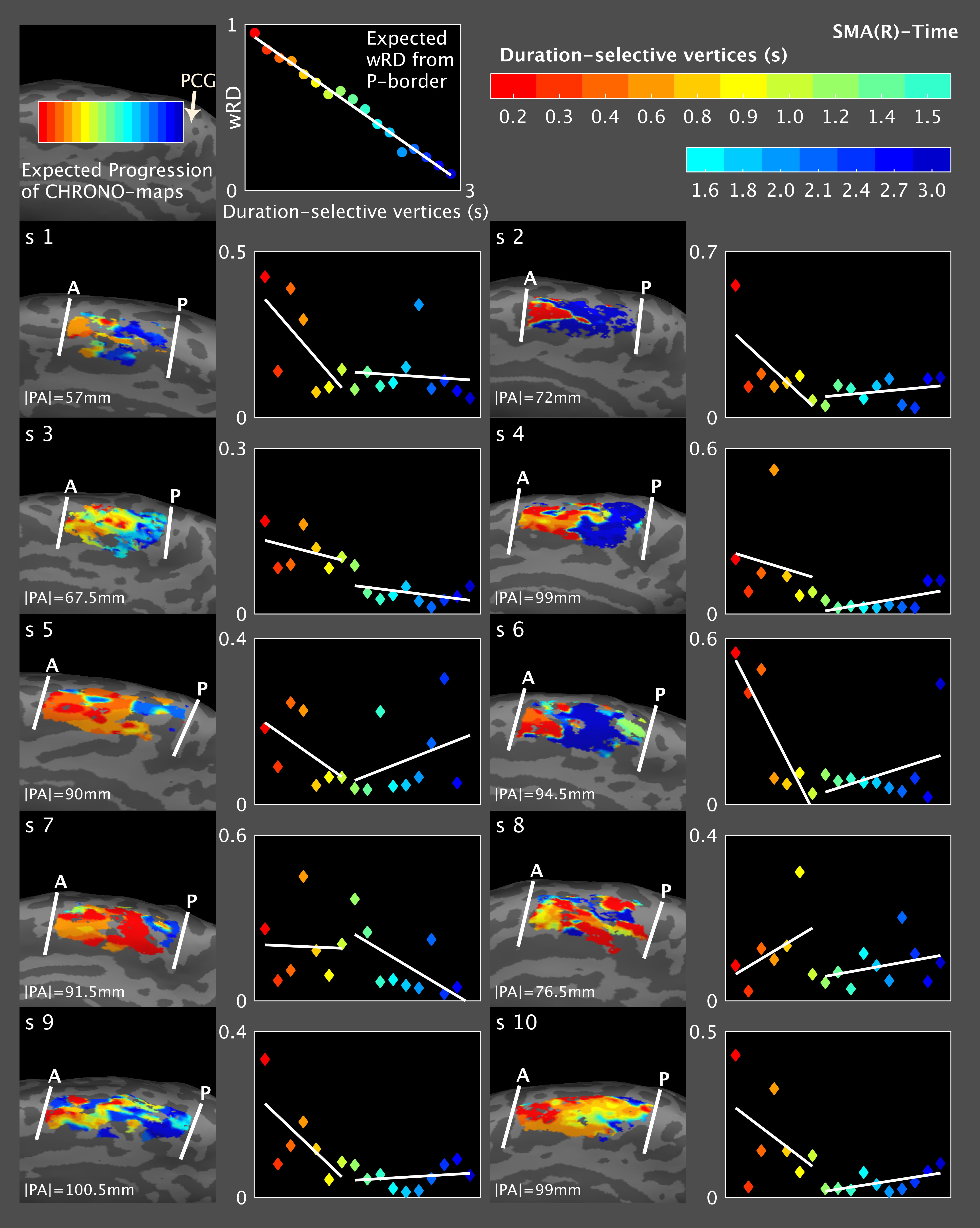

Supplement: S10 Fig — For the description of the figure see legend S8 Fig. The data can be found in S4 Data. fMRI, functional magnetic resonance imaging; L, left; SMA, supplementary motor area. (TIF) [file pbio.3000026.s011.tif]

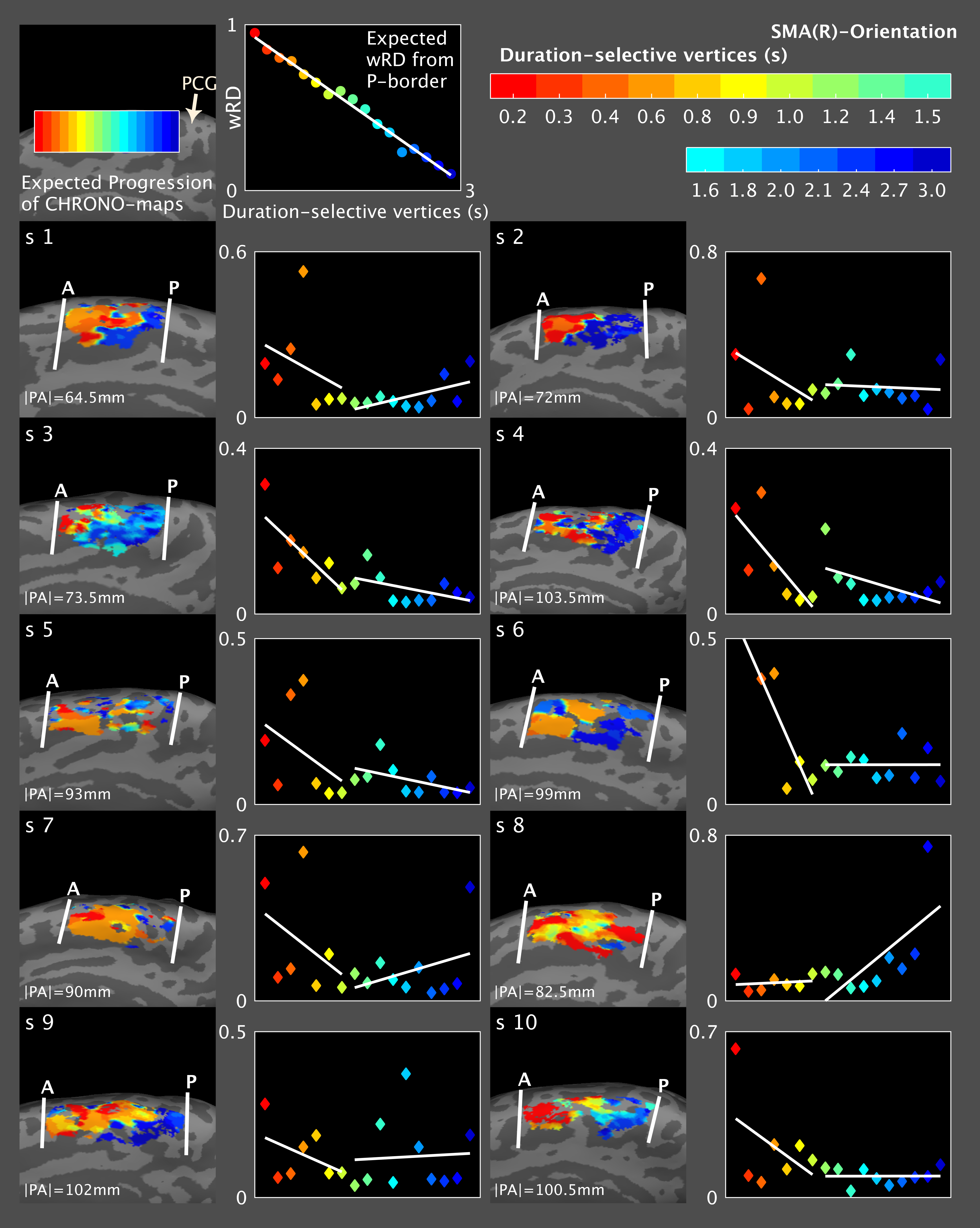

Supplement: S11 Fig — For the description of the figure, see legend S8 Fig. The data can be found in S4 Data. fMRI, functional magnetic resonance imaging; SMA, supplementary motor area. (TIF) [file pbio.3000026.s012.tif]

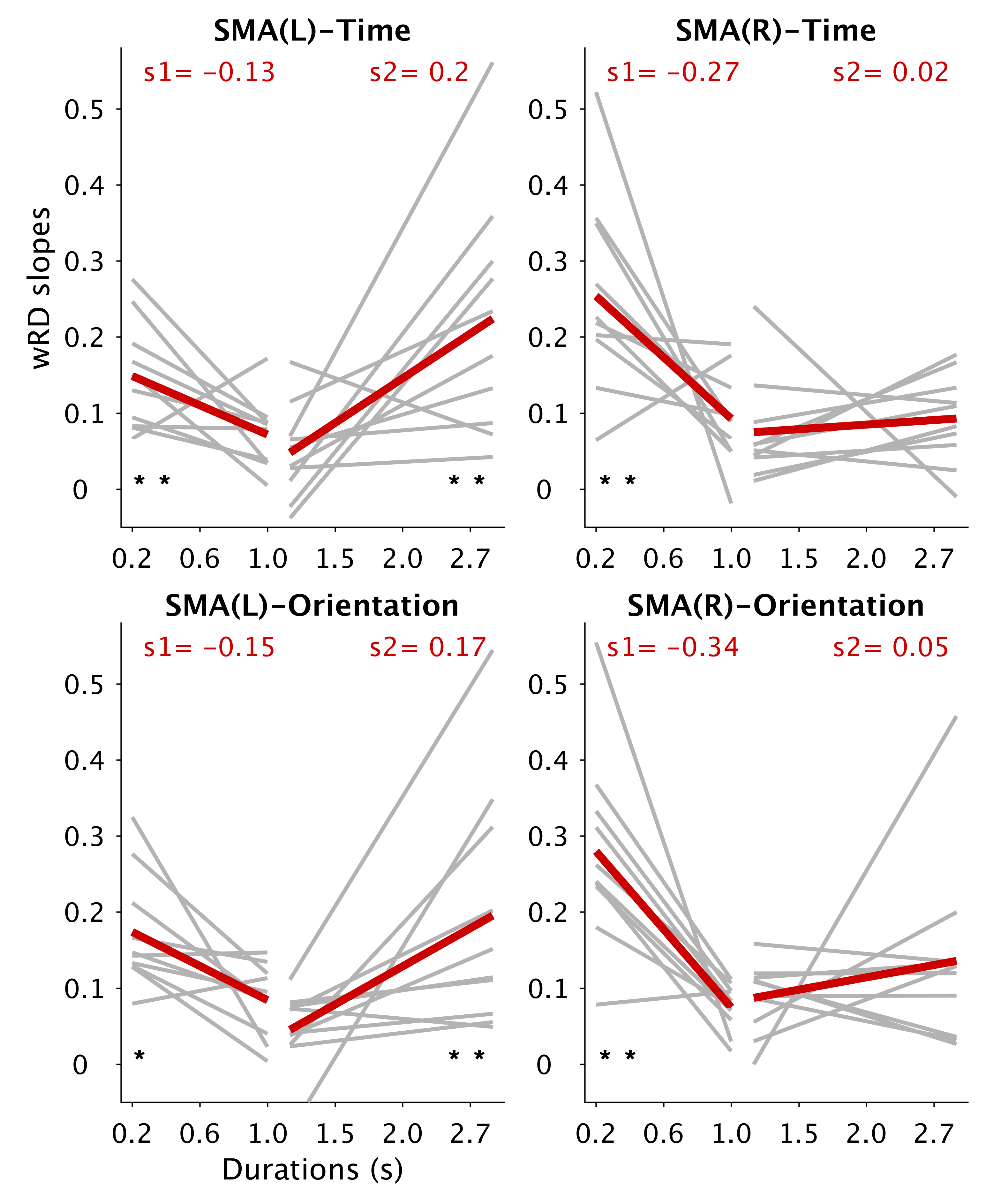

Supplement: S12 Fig — Individual slopes obtained by fitting the wRD from the posterior border of the 17 duration-selective clusters. For left and right SMA in the time and in the orientation task, we plotted the individual slopes (black lines) and the average slopes (red line). **P < 0.001, *P < 0.01. The data can be found in S3 Data. fMRI, functional magnetic resonance imaging; SMA, supplementary motor area; wRD, weighted relative distance. (TIF) [file pbio.3000026.s013.tif]

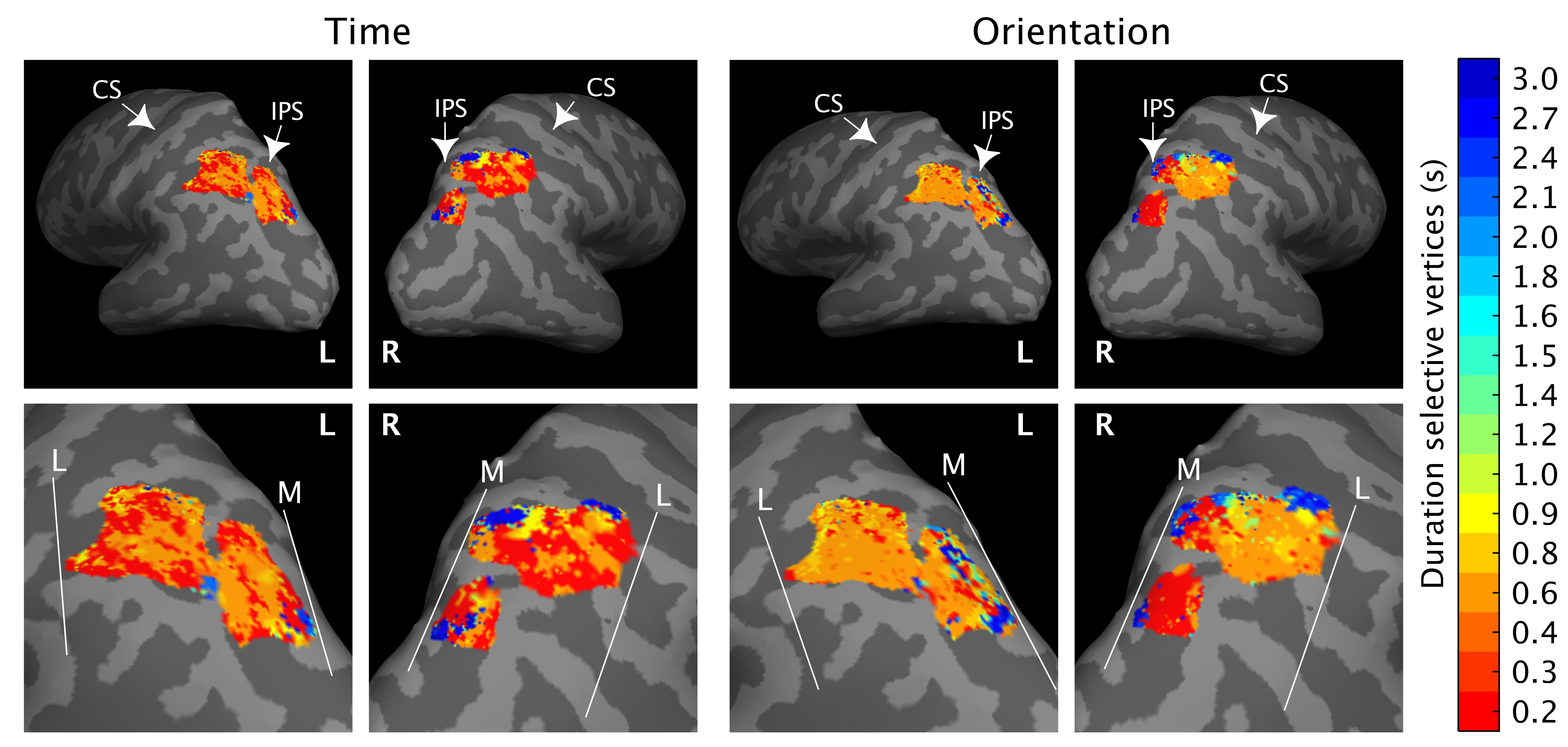

Supplement: S13 Fig — Here we show the estimated μ on the cortical surface for the IPS of both hemisphere of the estimated μ parameter. Different colors represent vertices (i.e., voxels projected onto the cortical surface) selective to different duration ranges (i.e., vertices with different estimated μ). We show the results of the group (average of 10 subjects) for the 17 estimated μ. The 17 μ are the 17 durations presented in the 10 different trial type (either S1 or S2). The color scale goes from red, i.e., shortest duration (0.2 s) to dark blue, i.e., longest duration (3 s). The white lines give an example of the map borders as they were drawn to estimate the wRD in the individual subjects. On the left-hand side, time maps in time task, on the right-hand side time maps in the orientation task. The data can be found in S4 Data. L, left; R, right; CS, central sulcus; IPS, intraparietal sulcus; L, lateral; M, medial; wRD, weighted relative distance. (TIF) [file pbio.3000026.s014.tif]

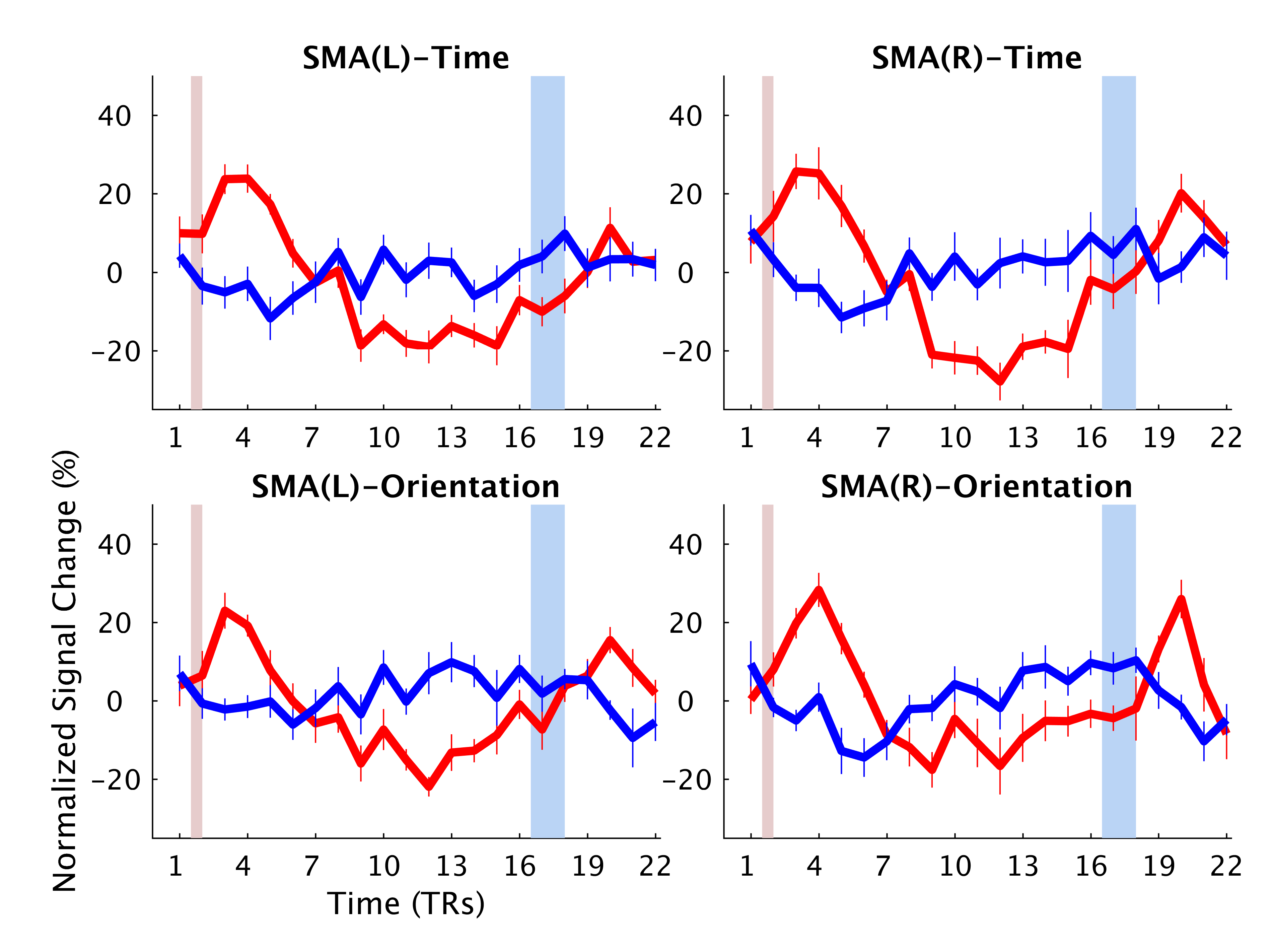

Supplement: S14 Fig — Normalized signal change of the shortest- (red line) and the longest- (blue line) duration–selective clusters of voxels over the time of a cycle (i.e., 44 seconds = 22 TRs) in left and right SMA for time and orientation tasks. For each subject, we averaged the signal across 20 cycles. Please note that the signal in descending cycles was swapped to match the ascending ones. The data can be found in S3 Data. BOLD, blood oxygenation level-dependent; SMA, supplementary motor area; TR repetition time. (TIF) [file pbio.3000026.s015.tif]

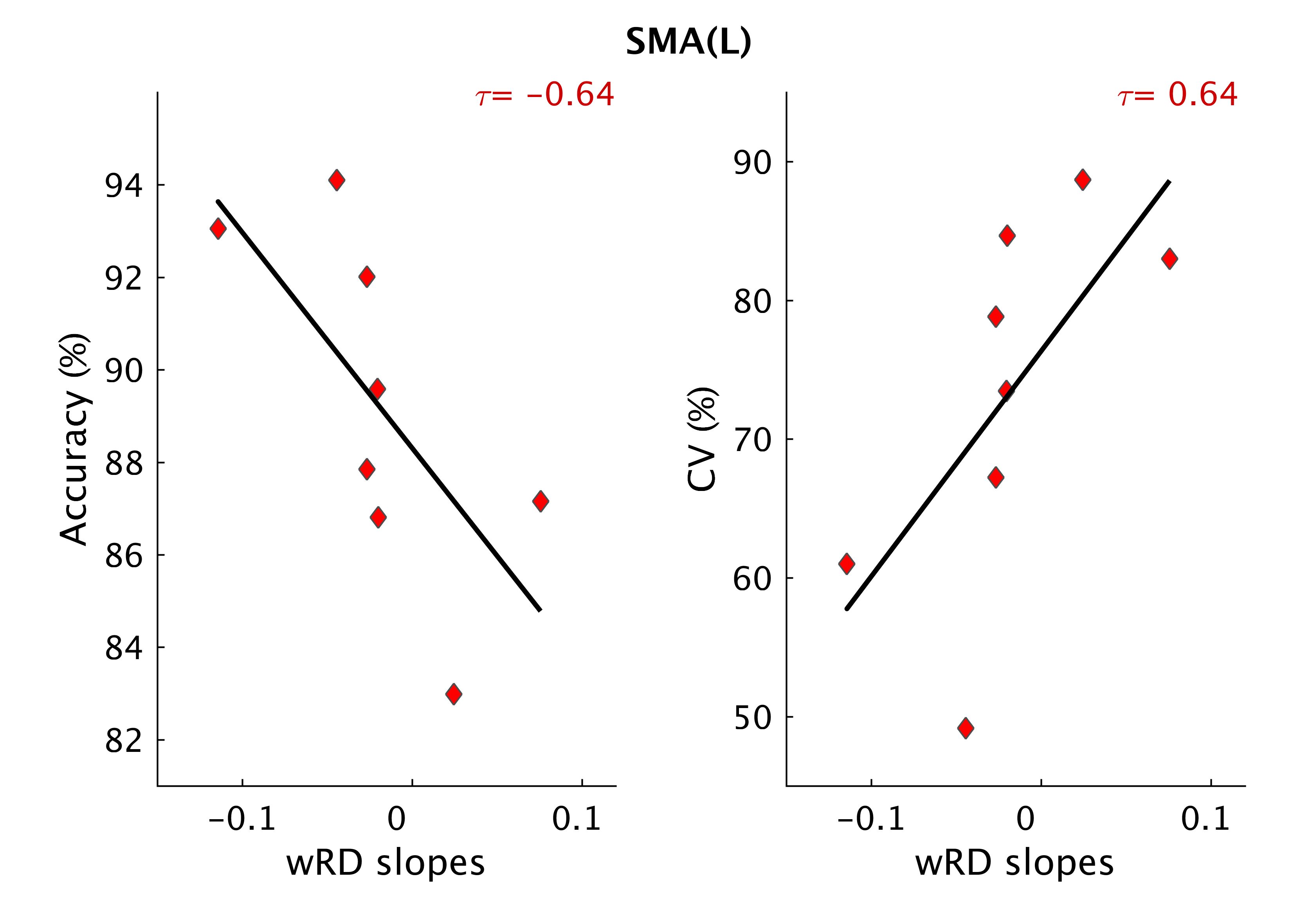

Supplement: S15 Fig — The two scatterplots show the correlations (using Kendal's tau correlation coefficient) between the individual slopes of the wRD in left SMA with two behavioral indexes of temporal performance: accuracy (left panel) and coefficient of variation (i.e., CV = standard deviation/duration, right panel). The data can be found in S3 Data. SMA, supplementary motor area; wRD, weighted relative distance. (TIF) [file pbio.3000026.s016.tif]

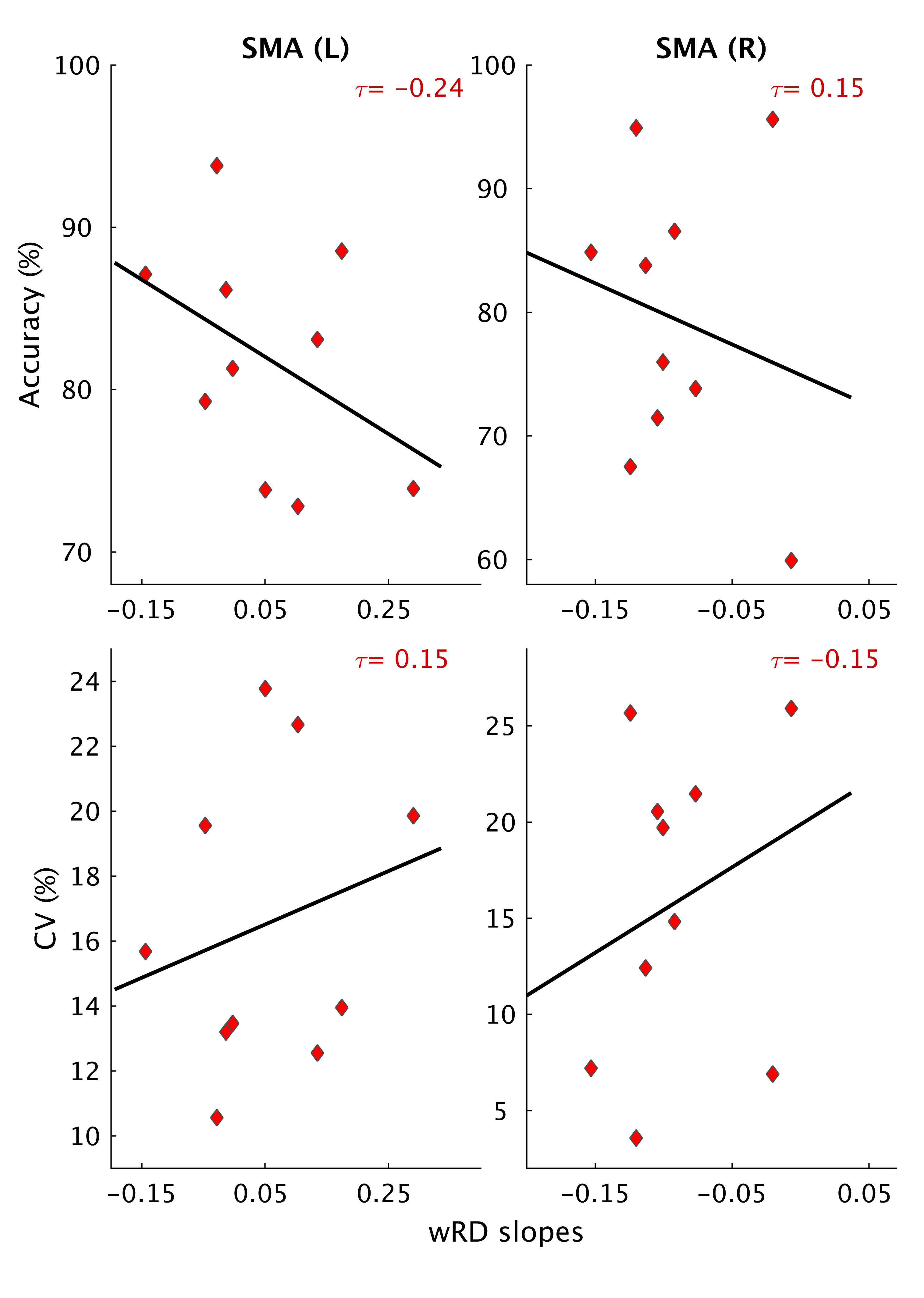

Supplement: S16 Fig — The scatterplots show the correlations (using Kendal's tau correlation coefficient) between the individual slopes of the wRD measured for left and right SMA, with two behavioral indexes of temporal performance: accuracy (upper panel) and coefficient of variation (i.e., CV = standard deviation/duration, lower panel). The data can be found in S3 Data. SMA, supplementary motor area; wRD, weighted relative distance. (TIF) [file pbio.3000026.s017.tif]

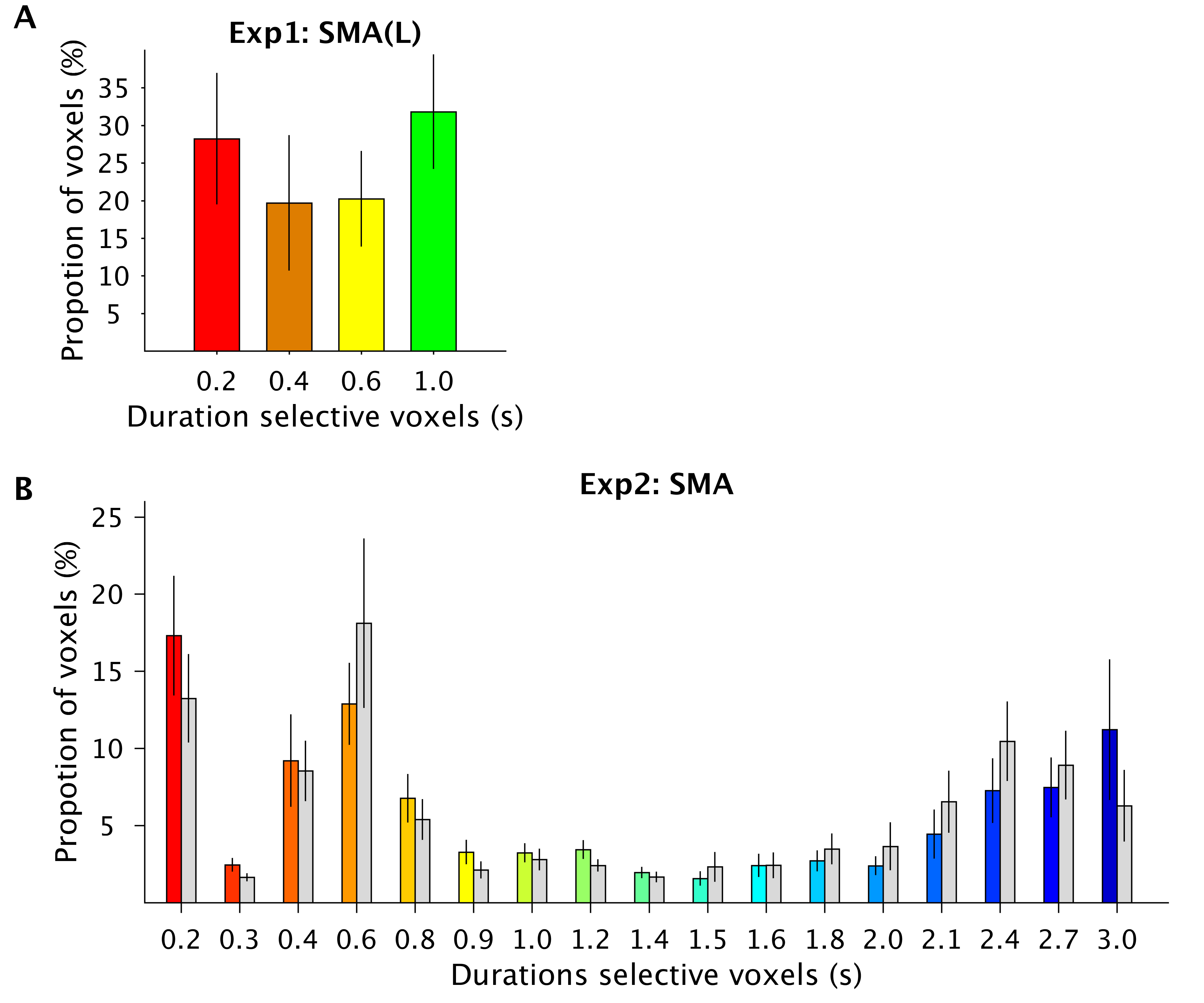

Supplement: S17 Fig — For both experiments, we show the mean and standard error of the proportions (i.e., number of vertices of a given type/total number of vertices in the map) of different duration-selective vertices within the SMA chronomaps. For Exp 1, we show SMA left (A). For Exp 2, we show the average of left and right SMA maps for the time and the orientation tasks (B). The data can be found in S3 Data. SMA, supplementary motor area (TIF) [file pbio.3000026.s018.tif]

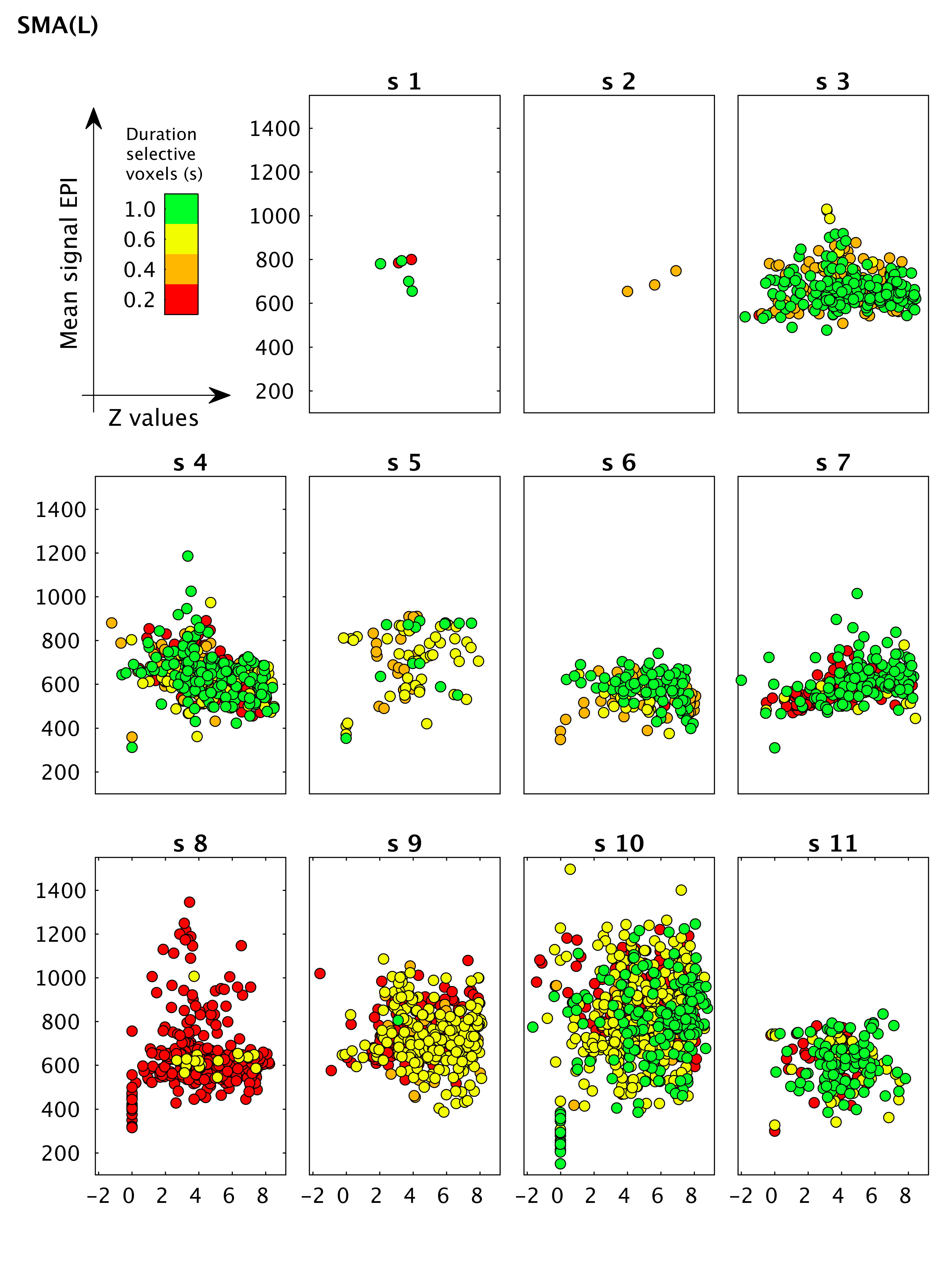

Supplement: S18 Fig — The scatterplots show for each subject and each duration-selective voxel the z scores (x-axis) plotted against the mean signal intensity across the 18 runs. None of the voxels had a very high z score and a very low signal intensity. The data can be found in S4 Data. (TIF) [file pbio.3000026.s019.tif]

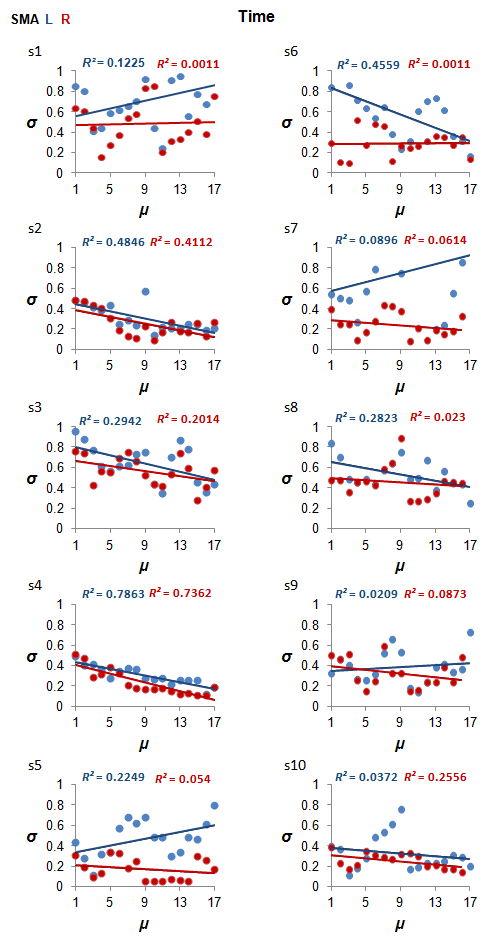

Supplement: S19 Fig — The plots show the correlations between σ and μ for the left (blue) and the right (red) SMA. Each plot is a subject. None of the voxels has a very wide spread; actually, all σ are <1. The data can be found in S3 Data. SMA, supplementary motor area. (TIF) [file pbio.3000026.s020.tif]

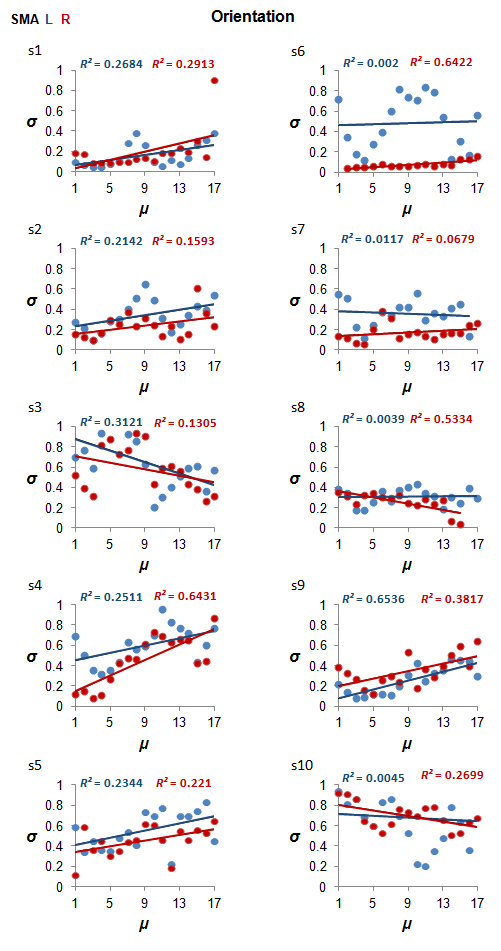

Supplement: S20 Fig — The plots show the correlations between σ and μ for the left (blue) and the right (red) SMA. Each plot is a subject. None of the voxels has a very wide spread (all μ are <1). The data can be found in S3 Data. SMA, supplementary motor area. (TIF) [file pbio.3000026.s021.tif]
